# Ion-channel regulation of response decorrelation in a heterogeneous multi-scale model of the dentate gyrus

**DOI:** 10.1016/j.crneur.2021.100007

**Published:** 2021-03-05

**Authors:** Poonam Mishra, Rishikesh Narayanan

**Affiliations:** Cellular Neurophysiology Laboratory, Molecular Biophysics Unit, Indian Institute of Science, Bangalore 560012, India

**Keywords:** Adult neurogenesis, Channel decorrelation, Computational model, Heterogeneities hippocampus, Intrinsic plasticity, Ion channels, Multiscale analysis

## Abstract

Heterogeneities in biological neural circuits manifest in afferent connectivity as well as in local-circuit components such as neuronal excitability, neural structure and local synaptic strengths. The expression of adult neurogenesis in the dentate gyrus (DG) amplifies local-circuit heterogeneities and guides heterogeneities in afferent connectivity. How do neurons and their networks endowed with these distinct forms of heterogeneities respond to perturbations to individual ion channels, which are known to change under several physiological and pathophysiological conditions? We sequentially traversed the ion channels-neurons-network scales and assessed the impact of eliminating individual ion channels on conductance-based neuronal and network models endowed with disparate local-circuit and afferent heterogeneities. We found that many ion channels differentially contributed to specific neuronal or network measurements, and the elimination of any given ion channel altered several functional measurements. We then quantified the impact of ion-channel elimination on response decorrelation, a well-established metric to assess the ability of neurons in a network to convey complementary information, in DG networks endowed with different forms of heterogeneities. Notably, we found that networks constructed with structurally immature neurons exhibited functional robustness, manifesting as minimal changes in response decorrelation in the face of ion-channel elimination. Importantly, the average change in output correlation was dependent on the eliminated ion channel but invariant to input correlation. Our analyses suggest that neurogenesis-driven structural heterogeneities could assist the DG network in providing functional resilience to molecular perturbations.

## 1 Introduction

A multitude of experimental and computational studies have established the role of the dentate gyrus (DG) as a brain region that is critically involved in memory encoding. Prominent among these encoding tasks is the ability of the DG to mediate response decorrelation and pattern separation of inputs received from the entorhinal cortex Li et al. (2017); Lodge and Bischofberger (2019); Aimone et al. (2014); Aimone et al. (2006); Aimone et al. (2009); Sahay et al. (2011); Leutgeb et al. (2007); Kropff et al. (2015); Mishra and Narayanan (2019); Marr (1971); Rolls (2013); Luna et al. (2019). In addition, the DG has also implicated in engram formation, which involves activity-dependent plasticity in neural excitability involving changes in ion channels in these neural populations (Titley et al., 2017; Tonegawa et al., 2015, 2018; Mozzachiodi and Byrne, 2010; Zhang and Linden, 2003). There are precise sets of computation spanning different scales of analyses that occur within the DG network towards accomplishing these physiological goals. Perturbations to components that drive these computations at one scale (say individual ion channel densities), introduced by pathological insults or neuromodulation or learning- or adaptation-induced changes, could result in a cascading effect that alters physiological properties across several scales (say, single neuron and network level outcomes). For instance, the requirement of DG neurons to change their ion channel densities (at the molecular scale) in the process of encoding engrams could alter their ability to perform response decorrelation (at the network scale). The complexity involved in the assessment of such multi-scale cascades is enormous, owing to the disparate forms of biological heterogeneities inherent to the different network components and the intricate interactions between these distinct components that govern network function. Therefore, computational models spanning different scales, where a systematic analyses of such cascades can be rigorously accomplished, provides a pragmatic path to approach this problem of multi-scale analyses in DG network physiology.

An essential first step in assessing the cascading impact of altered molecular components to multi-scale functions is to account for the expression of multiple forms of biological heterogeneities in the DG, spanning different scales of analysis. Biological heterogeneities that span the DG at single neuron scale include those in ion channel properties and expression profiles, neuronal intrinsic properties, dendritic arborization, whereas at the network scale it comprises mainly of local synaptic connectivity and the sparse, orthogonal and divergent nature of afferent connectivity. Importantly, each of these heterogeneities is further amplified by the expression of adult neurogenesis in the DG, especially given the enormous dependence of each neuronal and synaptic attribute on the neuronal maturation process. Especially with reference to afferent connectivity, accumulating lines of evidence point to adult neurogenesis providing a unique substrate for orthogonal, non-overlapping processing and storage of information from upstream cortex (Li et al., 2017; Lodge and Bischofberger, 2019; Aimone et al., 2006, 2009; Luna et al., 2019), referred to as afferent heterogeneities. The expression of these amplified heterogeneities, in conjunction with specific characteristics of the local network provide substrates for the prevalent postulates and lines of evidence that the DG network endowed with adult neurogenesis is an ideal system to execute response decorrelation and pattern separation (Li et al. (2017); Lodge and Bischofberger (2019); Aimone et al. (2014); Aimone et al. (2006); Aimone et al. (2009); Sahay et al. (2011); Leutgeb et al. (2007); Kropff et al. (2015); Mishra and Narayanan (2019); Mishra and Narayanan (2020b)).

To quantify the impact of molecular perturbation on network function, a critical second step is to employ a quantitative metric to monitor network function. Channel decorrelation is a type of response decorrelation, and is defined as a reduction in correlation between response profiles of individual channels (neurons) to afferent stimuli. Channel decorrelation has been identified as a mechanism to ensure that information conveyed by different neuronal channels is complementary (Mishra and Narayanan, 2019; Wiechert et al., 2010; Padmanabhan and Urban, 2010; Chow et al., 2012; Pitkow and Meister, 2012; Tetzlaff et al., 2012). It has been established that channel decorrelation could be achieved through synergistic interactions between different forms of these heterogeneities, with afferent heterogeneities dominating local heterogeneities when coexpressed (Mishra and Narayanan, 2019). In a scenario where afferent heterogeneities, actively mediated by afferent connectivity driven by adult neurogenesis (Li et al., 2017; Lodge and Bischofberger, 2019; Aimone et al., 2006, 2009; Luna et al., 2019), are dominant (Mishra and Narayanan, 2019), what is the precise role of local heterogeneities in the network? How does channel decorrelation vary in a network endowed with various local heterogeneities when neurons in the network encounter single ion-channel perturbations? Would the expression of specific forms of local heterogeneities in general, and neurogenesis-induced heterogeneities in particular, contribute to functional resilience of the perturbed network?

To understand the cascading impact of molecular-scale perturbations on cellular (neural integration and excitability) and network (channel decorrelation) physiology with a specific goal to understand the contributions of local heterogeneities to functional resilience, we employed a multi-scale conductance-based network model of the DG. The neurons of this network model were biophysically and physiologically constrained to match their biological counterparts, and the network was endowed with four distinct forms of local and afferent heterogeneities. We employed this model to sequentially assess the cascading impact of eliminating individual ion channels from two distinct neuronal subtypes, first on neuronal intrinsic physiological properties, and consequently on network excitability and on the ability of the network to perform channel decorrelation.

At the single-neuron scale, our analyses revealed that the mapping between ion channels and physiological measurements was many-to-many, but not all-to-all. Specifically, we found that many (but not all) ion channels differentially contributed to specific neuronal or network measurements, and the elimination of any given ion channel altered several (but not all) functional measurements. At the network scale, the impact of eliminating individual ion channels was critically reliant on the specific local heterogeneities expressed in the DG network. Importantly, in the presence of structurally immature neurons in the DG network, the impact of ion channel elimination on channel decorrelation was lower, when compared with a network exclusively constructed with structurally mature neurons. Finally, we observed that for perturbation in a given ion channel, the average percentage change in output correlation was invariant to the specific values of input correlation. Together, these results unveil the importance of ion-channel and neurogenesis-induced heterogeneities in maintaining robustness of channel decorrelation, emphasizing their role beyond providing a substrate (Mishra and Narayanan, 2019) for the expression of degeneracy in achieving channel decorrelation.

## 2 Methods

In this multi-scale computational study, we sought to systematically examine the impact of knocking out different ion channels on single neuronal response properties and on network-scale decorrelation in the DG. An important question addressed in our analyses is on whether and how the expression of local heterogeneities in the DG contributes to functional resilience (with reference to response decorrelation) in the face of ion channel perturbations. In what follows, we describe the single neuronal and network models, the procedures and measurements employed in assessing single-neuron and network physiology after virtual knockout of specific ion channels. To avoid confusion between the use of the word “channel” in channel decorrelation and ion channels, we always employ the phrase “ion channels” when we refer to the latter.

### 2.1 Rationale and experimental design: the need for the incorporation of heterogeneities in studying ion-channel perturbations

Analyzing the impact of individual ion channels on neuronal intrinsic properties in a single hand-tuned model introduces biases that are inherent to the specific model and would not account for the heterogeneities in ion channel expression or in intrinsic properties of the neurons. With the ubiquitous expression of ion-channel degeneracy, whereby synergistic interactions among disparate combinations of ion channels result in the emergence of similar single neuron physiological characteristics (Mishra and Narayanan, 2019; Basak & Narayanan, 2018, 2020; Das et al., 2017; Drion et al., 2015; Jain and Narayanan, 2020; Migliore et al., 2018; Mittal and Narayanan, 2018; Mukunda and Narayanan, 2017; Rathour et al., 2016; Rathour and Narayanan, 2012a, 2014, 2019; Anirudhan and Narayanan, 2015; Seenivasan and Narayanan, 2020; Srikanth and Narayanan, 2015), such an approach would yield results that are not applicable to the entire population of neurons in the biological system (Marder and Taylor, 2011). A well-established alternate to this approach, which accounts for degeneracy and heterogeneities across scales, is an unbiased stochastic search algorithm that spans the ion channel parametric space to arrive at neuronal models that satisfy cellular-scale physiological constraints (Prinz et al., 2003, 2004; Foster et al., 1993). As the population of neuronal models arrived through such an approach is well constrained by the biophysical and physiological measurements from the specific neuronal subtype under consideration, this population constitutes an efficacious substrate to understand the impact of individual ion channels on neuronal physiology. An important difference between a hand-tuned model and this stochastic search approach is that the stochastic search model is unbiased, with no relationship in terms of *which* ion channel was introduced for matching *what* specific physiological property. Therefore, results arrived on the impact of individual ion channels *emerge* from the heterogeneous neuronal populations, without assignment of specific physiological purposes to individual ion channels (Mishra and Narayanan, 2019; Basak and Narayanan, 2018; Mittal and Narayanan, 2018; Rathour and Narayanan, 2012a, 2014, 2019; Srikanth and Narayanan, 2015; Marder and Taylor, 2011; Prinz et al., 2003; Foster et al., 1993; Goldman et al., 2001; Taylor et al., 2009).

### 2.2 Intrinsically heterogeneous population of single-neuronal model obtained through unbiased stochastic search

**Fig. 1:**
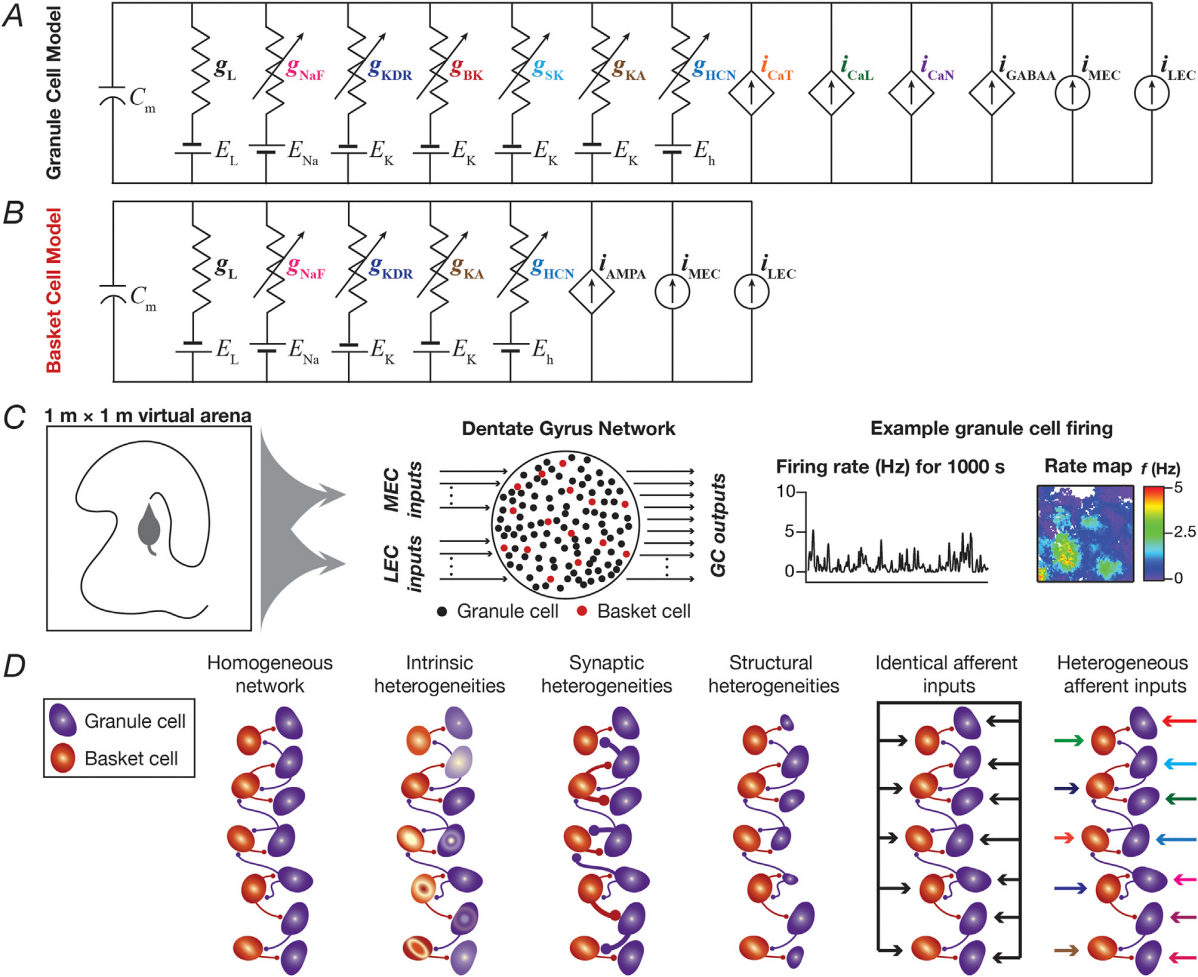
Multi-scale modeling framework for assessing the impact of ion channel knockouts on cellular and network physiology of the dentate gyrus during virtual arena traversal. *A-B*, Conductance-based models for granule cells (GC) and basket cells (BC) were built using several experimentally derived ion channel conductances for these neurons. Symbols employed: *g*_L_ Leak conductance; *g*_NaF_: Fast sodium conductance; *g*_KDR_: Delayed rectifier potassium conductance; *g*_BK_ Bigconductance calcium-activated potassium conductance; *g*_SK_: Small-conductance calcium-activated potassium conductance; *g*_BK_: *A*-type transient potassium conductance; *g*_HCN_: Hyperpolarization-activated cyclic-nucleotide-gated (HCN) nonspecific cation conductance; *i*_CaT_: *T*-type calcium current; *i*_CaL_: *L*-type calcium current; *i*_CaN_: *N*-type calcium current; *i*_GABAA_: GABAA receptor current; *i*_AMPA_: AMPA receptor current; *i*_MEC_: current from medial entorhinal cortical cells; *i*_LEC_: current from lateral entorhinal cortical cells. Note that all sodium, potassium and nonspecific cation channels were modeled using a Nernstian framework are represented as parallel conductances; all calcium currents and receptor currents were modeled using the Goldman-Hodgkin-Katz (GHK) formulation, and therefore are represented as dependent currents; the inputs from entorhinal cortex were modeled as currents that were dependent on animal traversal and are represented as current sources. *C*, A virtual animal was allowed to run in an arena of 1 m × 1 m (left panel) for a period of 1000 s to allow complete traversal of the entire arena. The animal’s location in this arena was fed into a dentate gyrus network made of interconnected granule and basket cells (middle panel). The voltage outputs of granule cells were recorded to obtain firing rate profiles and spatial rate maps (last panel) by overlaying neuronal firing rate over the temporally aligned spatial location of the virtual animal. *D*, The network employed here was not a homogeneous network, but employed several biological heterogeneities expressed in the dentate gyrus. Intrinsic heterogeneities represented variability in ion channel densities and neuronal intrinsic properties, and was accounted for by employing a multi-parametric multi-objective stochastic search (MPMOSS) paradigm (Mishra and Narayanan, 2019). Synaptic heterogeneities represented the strength of the local BC → GC and GC → BC connections, and were modeled by altering the AMPAR and GABAA receptor permeability. These receptor permeabilities were varied within a range where the excitation-inhibition balance was maintained and the overall firing rates of GCs and BCs were within experimentally observed ranges. Structural heterogeneities were introduced to model surface area changes in granule cells consequent to adult neurogenesis, and were incorporated by adjusting the geometry of the GC models. Afferent heterogeneities were representative of the uniquely sparse connectivity from the entorhinal cortices to the DG, and were modeled by feeding each GC and BC neuron with different afferent inputs. This scenario was compared with a case where all GCs and BCs were given *identical* afferent inputs.

We employed a multi-parametric multi-objective stochastic search (MPMOSS) algorithm as a route to generate a heterogeneous population of GC and BC neuronal models. In this study, to assess the impact of individual ion channels on neuronal and network physiology, we employed the valid models generated previously (Mishra and Narayanan, 2019). We briefly describe the details associated with the generation of these heterogeneous populations of single-neuron models. The stochastic search for valid granule cells involved 40 active parameters associated with passive properties, nine active conductances and calcium handling mechanisms (Supplementary Table S1; (Mishra and Narayanan, 2019)). The passive model parameters of granule cell were set as follows: the resting membrane potential, *V*_RMP_ = –75 mV; specific membrane resistance, *R*_m_ = 38 kΩ cm^2^; specific membrane capacitance, *C*_m_ = 1 μF/cm^2^; and the model cell was a cylinder of 63-μm diameter and 63-μm length. This resulted in passive charging time constant (*R*_m_ *C*_m_) to be 38 ms (Schmidt-Hieber et al., 2007) and passive input resistance (*R*_in_) of the cell to be 305 MΩ, matching the experimental value of 309 ± 14 MΩ (Chen, 2004). The nine different active conductances that were present in the GC model were (Fig. 1A): hyperpolarization-activated cyclic nucleotide gated (HCN or *h*), *A*-type potassium (KA), fast sodium (NaF), delayed-rectifier potassium (KDR), small conductance (SK) and big conductance calcium-activated potassium (BK), *L*-type calcium (CaL), *N*-type calcium (CaN) and *T*-type calcium (CaT). The ion channel kinetics and their voltage-dependent properties were adopted from experimental measurements from the GC (Magee, 1998; Beck et al., 1992; Ferrante et al., 2009; Aradi and Holmes, 1999). All calcium ion channels were modeled using the Goldman-Hodgkin-Katz (GHK) formulation (Goldman, 1943; Hodgkin and Katz, 1949), with default values of intracellular and extracellular calcium concentrations set as 50 nM and 2 mM, respectively. The evolution of cytosolic calcium concentration [*Ca*]*_c_*, was dependent on the current through voltage-gated calcium ion channels and involved a first order decay with a default calcium decay time constant, τ_Ca_ = 160 ms:

(1) $$\frac{d{\left[\text{Ca}\right]}_{c}}{dt}=-\frac{10000I_{Ca}}{36\cdot dpt\cdot F}+\frac{{\left[\text{Ca}\right]}_{\infty }-{\left[\text{Ca}\right]}_{\text{c}}}{\tau_{Ca}}$$

where *F* represented Faraday’s constant, τ_Ca_ defined the calcium decay constant in GCs (Eliot and Johnston, 1994), *dpt* = 0.1 μm was the depth of the shell into which calcium influx occurred, and [*Ca*]_∞_ = 50 nM is the steady state value of [*Ca*]_*c*_. In generating the physiologically-validated heterogeneous GC population, we subjected 20,000 unique models spanning a 40-parameter space (Table S1) to a validation procedure involving nine different single-cell electrophysiological measurements (Table 1) from GCs. We found 126 models (~0.63% of the total population) to be valid.

A similar MPMOSS strategy was employed to generate a heterogeneous population of basket cells (Fig. 1B), endowed with four different voltage-gated ion channels (HCN, KA, NaF and KDR), and involving a stochastic search space of 18 parameters (Table S2: ion channel and passive membrane properties (Mishra and Narayanan, 2019)). The passive parameters of the BC base model whose geometry was set as a cylinder with 66-μm diameter and 66-μm length were as follows: *V*RMP = –65 mV, *R*_m_ = 7.1 kΩ cm^2^, *C*_m_ = 1 μF/cm^2^. Here, we generated 8000 unique BC models, validated them against 9 electrophysiological measurements from BCs (Table 1) and found 54 valid BC models (~0.675% of the total population). The experimental bounds on measurements for granule and basket cells (Table 1) were obtained from (Aradi and Holmes, 1999; Krueppel et al., 2011; Lubke et al., 1998; Mott et al., 1997; Santhakumar et al., 2005). We have demonstrated ion channel degeneracy individually for the 126 valid GCs and for the 54 BCs (Mishra and Narayanan, 2019). We employ these valid model populations for the virtual knockout analyses, both at single-neuronal and network scales.

### 2.3 Subthreshold and suprathreshold physiological measurements employed to quantify the single-neuron properties

**Fig. 2:**
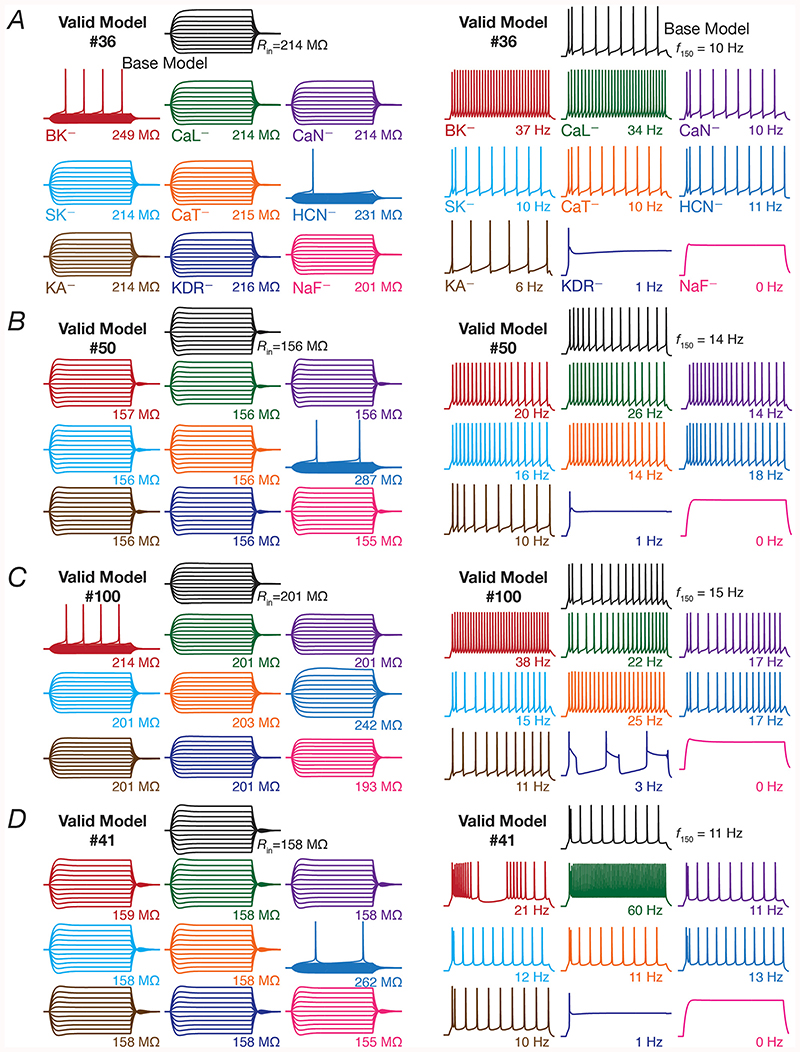
Virtual knockout of individual channels resulted in heterogeneous and differential impact on different sub- and supra-threshold electrophysiological measurements in a valid population of dentate granule cells. *A*, *Left*: Voltage responses to current pulses of –50 to +50 pA, in steps of 10 pA, for 500 ms employed for input resistance (*R*_in_) calculation. *Right*: Voltage traces showing firing rate f150) and spike pattern in response to a 150 pA current injection for 950 ms for the same granule cell model. *B-D*, Same as (*A*) but for different valid models of granule cell. Valid models 36 (*A*) and 50 (*B*), respectively represent the minimum and maximum percentage change in input resistance (Fig. 3G) after virtual knockout of HCN ion channels. Valid models 100 (*C*) and 41 (*D*), respectively represent the minimum and maximum percentage change in *f*_150_ (Fig. 3F) after virtual knockout of *L*-type calcium ion channels. Across panels, black traces represent the valid base model and traces of other colors depict those after virtual knockout of nine different ion channels in the chosen model.

The intrinsic response properties of GCs and BCs were quantified based on nine measurements (Mishra and Narayanan, 2019; Lubke et al., 1998), which were employed to validate the models obtained through stochastic search and to assess the impact of individual ion channel knockouts. The nine electrophysiological measurements employed for validation and their bounds are provided in Table 1. *R*_in_ was measured from the neuronal steady state voltage response to each of 11 different current pulses, injected with amplitudes ranging from –50 pA to 50 pA (for 1000 ms) in steps of 10 pA (*e.g*., Fig. 2A, left). The steady state voltage deflections from *V*RMP were plotted as a function of the corresponding current injections to obtain a *V–I* plot. We fitted a straight-line function to this *V–I* plot, and the slope of this linear fit defined *R*_in_. Sag ratio was calculated as the ratio of the steady state voltage deflection to the peak voltage deflection recorded in response to a –50 pA (1000 ms) current injection.

All supra-threshold measurements were obtained from the voltage trace recorded in response to a 150 pA depolarizing current injection, with AP measurements obtained from the first spike of this trace. Firing frequency was calculated as number of spikes in response to 150 pA current injection for 1 s (*e.g*., Fig. 2A, right). Spike frequency adaptation (SFA) was calculated as the ratio of the first inter spike interval (ISI) to the last ISI. The voltage in the AP trace corresponding to the time point at which the d*V*/d*t* crossed 20 V/s defined AP threshold. AP half-width was the temporal width measured at the half-maximal points of the AP peak with reference to AP threshold. AP amplitude was computed as the peak voltage of the spike relative to *V*_RMP_. Fast afterhyperpolarization (*V*_fAHP_) was measured as the maximal repolarizing voltage deflection of the AP from threshold (Mishra and Narayanan, 2019).

### 2.4 Virtual knockout approach and metrics employed to assess the impact of ion-channel knockouts on single-neuronal physiology

As disparate parametric combinations yielded similar physiological properties in heterogeneous populations of GC and BC models (Mishra and Narayanan, 2019), it was important to independently assess the impact of ion channel elimination in each of the 126 GCs and 54 BCs. Within the degeneracy framework, virtual knockout models (VKMs) constitute a powerful technique to quantitatively assess the contribution of specific ion channels to chosen measurements in a heterogeneous population of models (Basak & Narayanan, 2018, 2020; Jain and Narayanan, 2020; Mittal and Narayanan, 2018; Mukunda and Narayanan, 2017; Anirudhan and Narayanan, 2015; Rathour and Narayanan, 2014; Seenivasan and Narayanan, 2020). Specifically, for the GC population, we virtually knocked-out one of the 9 active ion channels (by setting its conductance value to be zero) individually from each of the 126 valid models, and computed each of the 9 measurements after this knockout. Then, we computed the percentage change in each of 9 measurements from their respective valid base model values (where all the ion channels were intact in that specific valid model). This procedure was repeated for all nine ion-channels in GCs, and the statistics of percentage changes in each measurement for each VKM were assessed. A similar procedure was applied independently on BC valid models as well, with differences in number of active ion channels in the model (*N*_channel_ = 4) and number of valid models (*N*_valid_ = 54). The procedure was employed to assess each of the 9 measurements here as well.

Quantitatively, for each of the 9 different measurements, let *M*_n_ (*base*) represent the measurement value of the base version (*i.e*., all parameters were intact) of model number *n* (1 ≤ *n* ≤ 126 for GCs; 1 ≤ n ≤ 54 for BCs). Let *M*_n_ (*C*_i_) represent the measurement value obtained from the VKM *after* deleting one the ion channels *C*_i_ (1 ≤ *i* ≤ 9 for GCs; 1 ≤ *i* ≤ 4 for BCs). We quantified the impact of single ion channel knockout on each measurement by computing percentage change as:

(2) $$\text{Δ}M_{n}\left(C_{i}\right)=\frac{M_{n}\left(C_{i}\right)-M_{n}(\text{base})}{M_{n}(\text{base})}\times 100$$

**Fig. 3:**
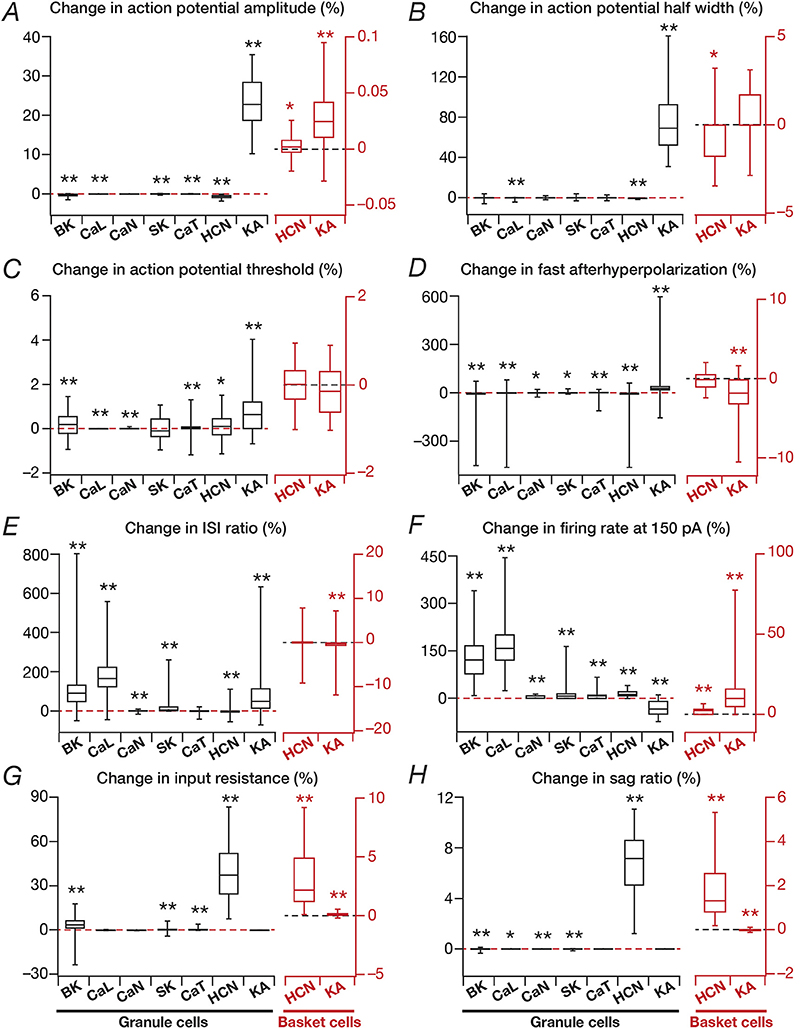
The mapping between individual ion channels and different electrophysiological measurements was many-to-many, with virtual knockout of individual ion channels yielding differential and variable effects on different measurements. *A-H*, Plots of percentage changes in different electrophysiological measurements obtained after virtual knockout of individual ion channels from valid models of granule (*N*_valid_ = 126 for GC, black) and basket (*N*_valid_ = 54 for GC, red) cell population obtained using MPMOSS. Percentage changes were calculated by comparing the measurement after virtual knockout of the specific ion channel with the measurement in the corresponding base model. Individual panels represent the following intrinsic measurements: *A*, action potential amplitude, *V*_AP_; *B*, action potential half-width, *T*_APHW_; *C*, action potential threshold, *V*th; *D*, fast after hyperpolarization potential, *V*_AHP_; *E*, ISI ratio; *F*, firing rate in response to 150 pA current injection, *f*_150_; *G*, input resistance, *R*_in_; and *G*, sag ratio. *p* values were obtained using Wilcoxon signed rank test, where the percentage change in measurements were tested for significance from a “no change” scenario. *: *p* < 0.01, **: *p* < 0.001.

This procedure was repeated for each valid model, each ion channel and each measurement, and the statistics of these measurements were plotted as quartiles to depict the entire span of changes (Fig. 3).

### 2.5 The multi-scale network and its local and afferent inputs

A default network of 100 GCs and 15 BCs (Fig. 1C), with the GC:BC ratio constrained by experimental observations (Aimone et al., 2009; Amaral et al., 2007), was constructed by randomly picking valid models from the population of GCs and BCs obtained from MPMOSS (Mishra and Narayanan, 2019). In a subset of simulations, the network size was increased to 500 GCs and 75 BCs, with similar model design (Fig. 8). Local connectivity was set such that the probability of a BC to GC connection was 0.1, and that of a GC to BC connection was set as 0.05 (Aimone et al., 2009). The GC → BC and BC → GC connections were modeled as synapses containing AMPA and GABA_A_ receptors, respectively. The AMPA receptors were modeled to be permeable to sodium and potassium ions, whereas the GABA_A_ receptors were permeable to chloride ions. Both receptor currents were modeled using the GHK convention (Goldman, 1943; Hodgkin and Katz, 1949), with a rise time of 2 ms and a decay time constant of 10 ms (Mishra & Narayanan, 2015, 2019).

(3) $$I_{AMPA}(v,t)=I_{AMPA}^{Na}(v,t)+I_{AMPA}^{K}(v,t)$$

where,

(4) $$I_{AMPA}^{\text{Na}}(v,t)={\bar{P}}_{\text{AMPA}}P_{Na}s(t)\frac{vF^{2}}{RT}\left(\frac{{\left[Na\right]}_{i}-{\left[Na\right]}_{o}\exp\left(-\frac{vF}{RT}\right)}{1-\text{exp}\left(-\frac{vF}{RT}\right)}\right)$$

(5) $$I_{\text{AMPA}}^{K}(v,t)={\bar{P}}_{\text{AMPA}}\;P_{K}\;s(t)\frac{vF^{2}}{RT}\left(\frac{{\left[K\right]}_{i}-{\left[K\right]}_{o}\exp\left(-\frac{vF}{RT}\right)}{1-\exp\left(-\frac{\mathrm{vF}}{\mathrm{RT}}\right)}\right)$$

where *F* was Faraday’s constant, *R* depicted gas constant, *T* was temperature and $\bar{P}$ *_AMPA_* was the maximum permeability of AMPAR. *s*(*t*) governed the AMPAR kinetics and was set as follows:

(6) $$s(t)=a\left(\exp\left(-t/\tau_{d}\right)-\exp\left(-t/\tau_{r}\right)\right)$$

where *a* normalized *s*(*t*) such that 0 ≤ *s*(*t*) ≤ 1, τ_d_ (=10 ms) represented the decay time constant, τ_r_ (=2 ms) depicted the rise time, *P*_Na_=*P*_K_, [*Na*]_i_ = 18 mM, [*Na*]*_o_* = 140 mM, [*K*]_*i*_ = 140 mM, and [*K*]*_o_* = 5 mM, leading to the AMPAR reversal potential to be ~0 mV. The BC→ GC GABA_A_ receptor chloride current was modeled as (Mishra and Narayanan, 2015):

(7) $$I_{GABAA}^{Cl}(v,t)={\bar{P}}_{GABAA}s(t)\frac{vF^{2}}{RT}\left(\frac{{\left[Cl\right]}_{i}-{\left[Cl\right]}_{o}\text{exp}(vF/RT)}{1-\exp(vF/RT)}\right)$$

where $\bar{P}$ *_GABAA_* was the maximum permeability of GABA_A_ receptor. *s*(*t*) was identical to that for AMPAR. [*Cl*]*_i_* = 5 mM and [*Cl*]*_o_* = 98 mM.

All neurons in this DG network received inputs from two different regions of entorhinal cortex (EC): one from medial entorhinal cortex (MEC) grid cells that transmitted spatial information and another from lateral entorhinal cortex (LEC), which provides contextual information (Renno-Costa et al., 2010; Anderson et al., 2007). The firing of the MEC and LEC cells were driven by the position of a randomly traversing virtual animal in a 1 m × 1 m arena (Fig. 1C). Each DG neuron received *active* inputs (Mishra and Narayanan, 2019; Cayco-Gajic et al., 2017) from 5 different MEC cells and 5 different LEC cells, with input strength from MEC and LEC split equally. The current input from a single MEC grid cell to DG cells was modeled as a hexagonal grid function defined as a sum of three two-dimensional cosine functions (Solstad et al., 2006):

(8) $$\psi (x,y)=\frac{2}{3}\left(\frac{\cos\left(g_{1}\right)+\cos\left(g_{2}\right)+\cos\left(g_{3}\right)}{3}+\frac{1}{2}\right)$$

where (*x*, *y*) represented the position of the virtual animal in the arena, and *g*_1_, *g*_2_ and *g*_3_ were defined as:

(9) $$g_{1}=\frac{4\pi \lambda }{\sqrt{6}}\left[\left(\cos\left(\theta +\frac{\pi }{12}\right)+\sin\left(\theta +\frac{\pi }{12}\right)\right)\left(x-x_{0}\right)+\left(\cos\left(\theta +\frac{\pi }{12}\right)-\sin\left(\theta +\frac{\pi }{12}\right)\right)\left(y-y_{0}\right)\right]$$

(10) $$g_{2}=\frac{4\pi \lambda }{\sqrt{6}}\left[\left(\cos\left(\theta +\frac{5\pi }{12}\right)+\sin\left(\theta +\frac{5\pi }{12}\right)\right)\left(x-x_{0}\right)+\left(\cos\left(\theta +\frac{5\pi }{12}\right)-\sin\left(\theta +\frac{5\pi }{12}\right)\right)\left(y-y_{0}\right)\right]$$

(11) $$g_{3}=\frac{4\pi \lambda }{\sqrt{6}}\left[\left(\cos\left(\theta +\frac{3\pi }{4}\right)+\sin\left(\theta +\frac{3\pi }{4}\right)\right)\left(x-x_{0}\right)+\left(\cos\left(\theta +\frac{3\pi }{4}\right)-\sin\left(\theta +\frac{3\pi }{4}\right)\right)\left(y-y_{0}\right)\right]$$

where λ represented the grid frequency, θ depicted the grid orientation and *x*_0_, *y*_0_ were offsets in *x* and *y*, respectively. This hexagonal grid function was scaled to obtain the input from a single MEC cell, with the scaling performed to set the relative contribution of MEC and LEC to the DG cells. MEC cell inputs were distinct in terms of the grid frequency (λ: 2–6 Hz) and grid orientation (θ: 0–360°), each sampled from respective uniform distributions. This hexagonal grid function was scaled to set the relative contribution of MEC and LEC to DG cells. Each MEC cell input was distinct in terms of the grid frequency and grid orientation, each randomly sampled from respective uniform distributions.

For modeling LEC inputs to GCs and BCs, we tiled the arena into 25 squares (5 rows and 5 columns) and assigned different tiles to be active for different LEC inputs (Renno-Costa et al., 2010). Inputs from this LEC cell to the DG cell was then scaled to set equal relative contribution of MEC and LEC to the DG cells. Each LEC cell input was associated with a unique randomized matrix, representing different active and inactive regions (Mishra and Narayanan, 2019; Renno-Costa et al., 2010).

### 2.6 Incorporating different forms of heterogeneities into the multi-scale network

To assess the robustness of networks (in performing channel decorrelation) endowed with different forms of heterogeneities to single ionchannel knockouts, we built DG networks endowed with distinct combinations of four different types of heterogeneities (Fig. 1D), following the approaches introduced in (Mishra and Narayanan, 2019):

(i) *intrinsic heterogeneity*, where the GC and BC model neurons had widely variable intrinsic parametric combinations, yet yielded physiological measurements that matched their electrophysiological counterparts (Mishra and Narayanan, 2020b; Beck et al., 1992; Ferrante et al., 2009; Aradi and Holmes, 1999; Santhakumar et al., 2005). As mentioned in earlier sections, this was incorporated into the network by the use of independent MPMOSS algorithms for generating BC and GC models.
(ii) *synaptic heterogeneity*, where the synaptic strength of the local GC-BC network was variable with excitatory and inhibitory synaptic permeability values picked from uniform random distributions. These parameters were maintained at a regime where the peakfiring rate of GCs and BCs stayed within their experimental ranges of 4-10 Hz and 30-50 Hz, respectively (Leutgeb et al., 2007). We ensured that extreme parametric combinations where the cell ceased firing (because of depolarization-induced block at one extreme or high inhibition at the other) were avoided, implying a balance between excitatory and inhibitory connections.
(iii) *neurogenesis-induced structural heterogeneity*, where the DG network was constructed entirely of mature or immature neurons, or constructed from neurons that represented different randomized neuronal ages. Populations of immature GCs (originating through adult neurogenesis) were obtained by subjecting the mature set of the GC valid models (obtained through MPMOSS) to structural plasticity. Specifically, the reduction in dendritic arborization and in the overall number of ion channels expressed in immature neurons (Aimone et al., 2014; van Praag et al., 2002) was approximated by a reduction in the surface area (diameter) of the model neuron, using *R*_in_ as the measurement to match with experimental counterparts. The diameters of GC for the three distinct configurations were: fully mature (63 μm), fully immature (2–9 μm) and heterogeneous age population (2–63 μm). Neurogenesis-induced structural heterogeneity was confined to the GC population, leaving the BC population to be mature.
(iv) *input-driven or afferent heterogeneity*, where all neurons in the GC and BC populations received either *identical* inputs (absence of afferent heterogeneity) from the EC, or *each* GC and BC received unique inputs (presence of afferent heterogeneity) from the EC. Afferent heterogeneity models incorporate sparse and orthogonal afferent connectivity from the EC to the DG (Li et al., 2017; Lodge and Bischofberger, 2019; Aimone et al., 2006, 2009, 2010, 2011, 2014; Amaral et al., 2007; Aimone and Gage, 2011; Andersen et al., 2006).

We tested the impact of virtually knocking out individual ion channels in networks endowed with different combinations of these biological heterogeneities, to ensure that our conclusions were not reflections of narrow parametric choices and to ask *if* the expression of heterogeneities enhances the robustness of the network to ion channel perturbations. There are several lines of evidence that the synaptic connectivity to immature neurons are low, and that this low connectivity counterbalances their high excitability Li et al. (2017); Mongiat et al. (2009); Dieni et al. (2016). To account for these observations, we reduced the overall afferent drive in scenarios that involved neurogenesis-induced structural differences (*i.e*., the fully immature population or the heterogeneous age population). This reduction was implemented by scaling the afferent drive in a manner that was reliant on the neuronal diameter, with lower diameter translating to larger reduction in the synaptic drive, and was adjusted towards the goal of reducing firing rate variability across the neuronal population (Mishra and Narayanan, 2019).

### 2.7 Network analyses: virtual animal traversal and assessment of excitability and channel decorrelation

A virtual animal was allowed to traverse a 1 m x 1 m arena, and the *x* and *y* coordinates of the animal’s location translated to changes in the external inputs from the MEC and LEC. The direction (range: 0–360°) and distance per time step (velocity: 2.5–3.5 m/s) were randomly generated, and were updated every millisecond. The amount of time taken for the virtual animal to approximately cover the entire arena was ~1000 s. All simulations were performed for 1000 s, with the spatiotemporal sequence of the traversal maintained across simulations to allow direct comparisons, with the initial position set at the center of the arena. After the network was constructed with different forms of heterogeneities and/ or with different ion channels knocked out, the spike timings of each GC were recorded through the total traversal period of 1000 s (Mishra and Narayanan, 2019). Note that the impact of knocking out the spike generating conductances (from either BC or GC) was not assessed because firing rate or response correlation could not be computed without a spiking response from the neurons.

**Fig. 4:**
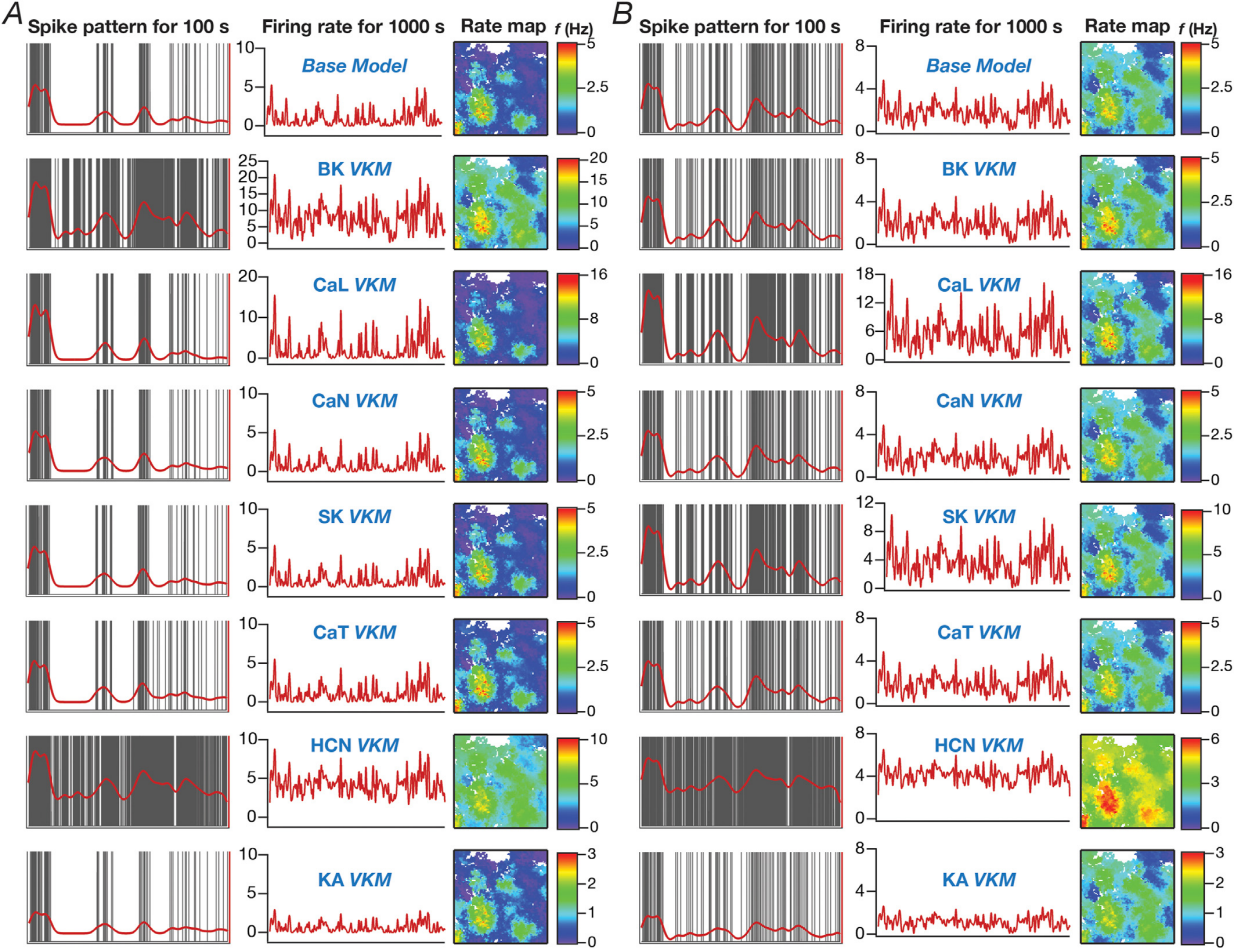
Granule cell firing profiles and spatial maps depicting the heterogenous impact of virtually knocking out individual ion channels from granule cells in a network receiving *identical* afferent inputs. *A, Left:* Spike patterns (gray) overlaid with firing rates (red) for a 100 s period for valid GC model 50, residing in a GC-BC network endowed with intrinsic and synaptic heterogeneities and receiving identical afferent inputs. *Center:* Instantaneous firing rates of GC model 50 for the entire 1000 s of animal traversal across the arena. *Right:* Color-coded spatial rate maps showing firing rate of GC model 50 superimposed on the trajectory of the virtual animal. The top-most panels represents these measurements for the base model (where all ion channels are intact), and the other panels depict these measurements obtained after virtual knockout of individual ion channels from the granule cell population of the network. *B*, Same as (*A*) for GC model 44 residing in the same network. Models 50 and 44 respectively showed maximum and minimum changes in firing rate after virtual knockout of BK ion channel (see Fig. 5A). The network employed in this illustrative example was endowed with intrinsic and synaptic heterogeneities, but did not express structural heterogeneities.

**Fig. 5:**
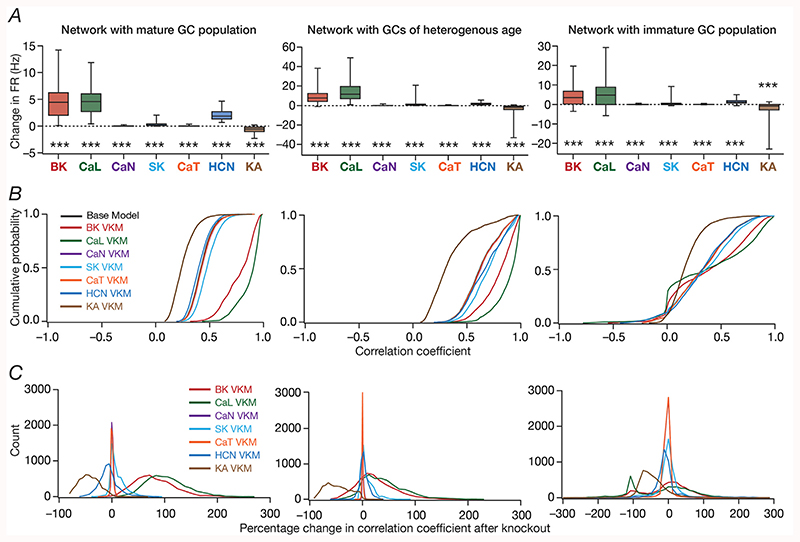
Virtual knockout of individual ion channels from granule cells resulted in differential and variable impact on channel decorrelation in networks endowed with distinct heterogeneities and receiving identical afferent inputs. *A*, Difference in firing rates (Eq. (12)) for all granule cells in the network, represented as quartiles. Firing rate of each cell was computed from the spike count of the cell for the entire 1000 s traversal of the virtual animal. p values were obtained using Wilcoxon signed rank test, where the change in firing rate was tested for significance from a “no change” scenario. ***: *p* < 0.001. *B*, Cumulative distribution of inter-neuronal pairwise firing rate correlation coefficients for networks built with either the base models, or with models after virtual knockout of individual ion channels. Shown are plots for the base model network and for the networks built with GC neurons where one of the 7 ion channels was virtually knocked out. *C*, Distribution of percentage changes in correlation coefficients for neuronal responses from the VKM network, compared to the respective base model coefficients. Shown are plots corresponding to percentage changes in networks built with GC neurons where one of the 7 ion channels was virtually knocked out. For (*A-C*), plots are shown for simulations performed with three distinct networks and associated virtual knockouts: network with a fully mature GC population (*left*), network with a GC population of heterogeneous age (*center*) and network with a fully immature GC population (*right*). Note that all three networks are endowed with intrinsic and synaptic heterogeneities. All neurons in the network received identical afferent inputs.

The overall firing rate of granule cells (*e.g*., Fig. 5A) spanning the 1000 s period was computed as the ratio between the spike count during the period and the total time (1000 s). Instantaneous firing rates for each GC was computed from binarized spike time sequences by convolving them with a Gaussian kernel with a default standard deviation (σ_FR_) of 50 ms. Although all correlation computations (*e.g*., Fig. 5B) were computed using a σ_FR_ of 50 ms, for displaying firing rate overlaid on the arena, we employed a smoother instantaneous firing rate computed with a σ_FR_ of 2 s (*e.g*., Fig. 4A). This is important because the choice of σ_FR_ plays a critical role in correlation computation (Mishra and Narayanan, 2019).

Histograms of Pearson’s pairwise correlation coefficients were computed between instantaneous firing rate arrays (each spanning the 1000 s period) of each GC. Specifically, a correlation coefficient matrix was constructed, with the (*i*, *j*)^th^ element of this matrix assigned to the Pearson’s correlation coefficient (*R*_ij_) computed between the instantaneous firing rate arrays of neuron *i* and neuron *j* in the network (channel decorrelation). As these correlation matrices are symmetric with all diagonal elements set to unity, we employed elements in the lower triangular part for computing the associated cumulative histogram (*e.g*., Fig. 5B).

Note that in this study, our focus is on channel decorrelation, a form of response decorrelation that is distinct from pattern decorrelation, where the focus in on correlation between temporally-aligned response profiles of individual ion channels of information (*i.e*., neurons) to afferent stimuli. Channel decorrelation is postulated to decrease the overlap between channel responses (*i.e*. individual neuronal responses), resulting in a code that is efficient because the information conveyed by different channels (*i.e*. other neurons) is largely complementary. This is distinct from pattern decorrelation, which is assessed by computing response correlations across these two sets of neuronal outputs when inputs corresponding to two different patterns arrive onto the same network. Pattern decorrelation is computed to determine the ability of neuronal outputs to distinguish (pattern separation) between the two input patterns (Mishra and Narayanan, 2019; Wiechert et al., 2010).

In assessing channel decorrelation as a function of input correlation (Mishra and Narayanan, 2019), we first computed the total afferent current impinging on each neuron. As the total current was the same for scenarios where identical afferent inputs were presented, the input correlation across all neurons was set at unity. For the scenario where the afferent inputs were heterogeneous, pairwise Pearson’ s correlation coefficients were computed for currents impinging on different DG neurons and were plotted against the corresponding response correlation (for the same pair). Output correlations in this plot were binned for different values of input correlation, and the statistics (mean ± SEM) of response correlation were plotted against their respective input correlation bins (*e.g*., Fig. 7A).

### 2.8 Network analyses: assessing the impact of the elimination of individual ion channels on network physiology

For each network configuration with any of the 9 ion-channels (7 from GCs and 2 from BCs) virtual knockouts, we computed change in this overall GC firing rate after virtual knockout of the ion channel. Quantitatively, let *F*_n_ (base) represent the overall firing rate of neuron *n* (1 ≤ *n* ≤ 100 for GC; 1 ≤ *n* ≤ 15 for BC) in the default network, and let *F*_n_ (*C*_i_) represent the firing rate of the same neuron, obtained from a network where one of the ion channels *C*_i_ (1 ≤ *i* ≤ 7 for GC VKMs; 1 ≤ *i* ≤ 2 for BC VKMs) was knocked out from all neurons (in either the GC or BC population). We quantified the impact of single ion channel knockout on firing as a difference:

(12) $$\text{Δ}F_{n}\left(C_{i}\right)=F_{n}\left(C_{i}\right)-F_{\text{n}}(\text{base})$$

This procedure was repeated for different network configurations endowed with different sets of heterogeneities. The statistics of these measurements were plotted as quartiles to depict the entire span of changes (*e.g*., Fig. 5A).

To compute pairwise changes in the degree of decorrelation consequent to ion channel knockout, we calculated percentage changes in the specific pairwise Pearson’s correlation coefficient after knockout of a specific ion channel, compared to the coefficient’s value before knockout. Quantitatively, let *R*_ij_ (base) represent the Pearson’s correlation coefficient computed between the instantaneous firing rate arrays of neuron *i* and neuron *j* (1 ≤ *i, j* ≤ 100; *i* ≠ *j*) in the base version of the network. Let *R*_ij_ (*C*_k_) represent the Pearson’s correlation coefficient computed between the same neuronal pair (*i*, *j*), obtained from a network where one of the ion channels *C*_k_ (1 ≤ *k* ≤ 7 for GC VKMs) was knocked out from all neurons (in either the GC or BC population). We quantified the impact of single ion channel knockout on output correlation of GCs as a percentage change:

(13) $$\text{Δ}R_{ij}\left(C_{k}\right)=\frac{R_{ij}\left(C_{k}\right)-R_{ij}(\text{base})}{R_{ij}(\text{base})}\times 100$$

This procedure was repeated for different network configurations endowed with different sets of heterogeneities. The statistics of these percentage changes were plotted as histograms (*e.g*., Fig. 5C), or were plotted against their respective input correlation values that were binned (*e.g*., Fig. 7B) using a procedure similar to the output correlation *vs*. input correlation plot mentioned above.

### 2.9 Computational details

All simulations were performed using the NEURON simulation environment (Carnevale and Hines, 2006), at 34 °C with an integration time step of 25 μs. Analysis was performed using custom-built software written in Igor Pro programming environment (Wavemetrics). Statistical tests were performed in statistical computing language R (www.R-project.org).

A relatively small network (compared to (Mishra and Narayanan, 2019)) was employed in this study owing to the computational complexity of the knockout simulations, each involving different network configurations, simulations and analyses, spanning the 7 ion-channel VKMs in GC and 2 ion-channel VKMs in BC, and for the expression or absence of different forms of heterogeneities. Simulations in this study were performed and results analyzed for a total of 40 ((base model + 7 GC VKMs + 2 BC VKMs) × (4 sets of heterogeneity configurations)) distinct network configurations, with each configuration entailing a period of 1000 s virtual traversal (with a simulation integration time step *dt* of 25 μs), accompanied by correlation and firing rate analyses spanning these large time series arrays. However, the impact of network size on our conclusions was assessed in a subset of simulations with networks that were constructed with 500 GCs and 75 BCs.

## 3 Results

The primary objective of this study was to address the question on whether and how the elimination of individual ion channels impact channel decorrelation in a DG network endowed with distinct forms of biological heterogeneities. The very nature of the question involved sequential traversal across three distinct scales of analyses (ion channels-neurons-network), and required that we account for the different biological heterogeneities expressed in the DG. Therefore, we built a multiscale DG network, which was constructed from a heterogeneous population of biophysically constrained and electrophysiologically validated conductance-based neuronal models for both GCs (Fig. 1A) and BCs (Fig. 1B). The afferent inputs to the DG network from the medial and lateral entorhinal cortices were driven by the position of a virtual animal traversing a 1 m × 1 m arena (Fig. 1C). We constrained the local excitatory-inhibitory connectivity and scaled the afferent inputs from the EC such that the firing rates of GCs and BCs matched their electrophysiological counterparts from *in vivo* recordings from awake-behaving animals. We recorded firing rates from all granule cells within the network and employed them for further analyses. This configuration provided us with an ideal setup to understand the impact of components in the molecular scale (ion channels) on functional outcomes (channel decorrelation) at the network scale, after rigorously accounting for cellular-scale physiological properties and how they emerge from interactions among disparate ion channels expressed at the molecular scale. We then employed appropriate techniques developed earlier (Mishra and Narayanan, 2019) to incorporate four distinct forms of heterogeneities into this network (Fig. 1D), to analyze the impact of eliminating individual ion channels on channel decorrelation in networks configured with different sets of these heterogeneities. As a first step in our analyses, we assessed the impact of virtually knocking out individual ion channels on single-neuron properties of the heterogeneous GC and BC populations.

### 3.1 Multiple ion channels differentially impact different single-neuron physiological features of granule and basket cells

The variable expression of a plethora of ion channels in individual neuronal subtypes, along with the interactional complexity involving other ion channels, bestows a neuron with its signature physiological characteristics. Whereas certain ion channels play a dominant role in *mediating* specific sub- and supra-threshold properties of neuron (*e.g*., action potential generation through an interplay between fast sodium and delayed-rectifier potassium ion channels), others play a regulatory role by *modulating* or *refining* fine details of neuronal physiology (*e.g*., modulation of neuronal firing rate by transient potassium ion channels). How do the 9 different active ion channels expressed in GCs and the 4 different active ion channels in BCs impact their respective physiological measurements?

To understand the contribution of these voltage-gated ion channels on single-neuron physiology of the heterogeneous populations of GCs and BCs, each independently exhibiting ion-channel degeneracy (Mishra and Narayanan, 2019), we turned to virtual knockout models. Here, each conductance in a given model was independently set to zero, and the 9 single-cell physiological measurements were recomputed after this virtual knockout of the ion channel (Fig. 2). We repeated VKMs for all the 126 models and 9 ion channels in GC population (Fig. 1A), and the 54 models and the 4 ion channels in BC population (Fig. 1B), spanning all the 9 physiological measurements for both populations (Fig. 3). As NaF and KDR ion channels didn’ t alter sub-threshold measurements significantly (examples in Fig. 2), and the absence of either of these ion channels resulted in loss of action potential firing or repolarization, we have not included these VKM results. In addition, as the firing rate of the neuron in response to 50 pA current changed only for a small proportion of models and knockouts, we have not incorporated that measurement into our results. Therefore, we analyzed 8 measurements each from VKMs for 7 ion channels in the GC population, and VKMs for 2 ion channels in the BC population (Fig. 3).

Examples of virtual knocking out each of the 9 ion channels in 4 different GC models are depicted in Fig. 2. These examples illustrate the *differential* and *variable* response of individual models to ion channel knockouts. Firstly, considering the example model #36 (Fig. 2A), we noticed that the changes observed in the sub-threshold (input resistance, Fig. 2A, *left*) and supra-threshold (firing rate for 150 pA pulse current injection, Fig. 2A, *right*) responses were *differential*, with reference to knocking out different ion channels. For instance, knocking out the CaN ion channels did not change the input resistance or firing rate whereas knocking out BK ion channels introduced large changes to input resistance and firing rate; but knocking out either NaF or KDR ion channels altered firing rate without altering input resistance significantly. These observations pointed to different measurements in the *same* model being *differentially* sensitive to different ion channel knockouts. Second, valid model #36 (Fig. 2A) and #50 (Fig. 2B), respectively represent the minimum (100 × (231–214)/214 = 7.9%) and maximum (100 × (287–156)/156 = 83.9%) percentage change in *R*_in_ after virtual knockout of HCN ion channels. Thus, whereas the contribution of HCN ion channels to *R*_in_ is low for model #36, the contribution is higher for model #50. However, the contribution of BK ion channels to *R*_in_ is high for model #36, whereas it is lower for model #50. This represents the *variability* in the dependence of different models on the *same* ion channel in regulating a given physiological measurement, and demonstrates that the contribution of a given structural component to a functional measurement is heterogeneous (Rathour and Narayanan, 2019). This observation is further emphasized by valid model #100 (Fig. 2C) and #41 (Fig. 2D), respectively representing the minimum (100 × (22–15)/15 = 46.6%) and maximum (100 × (60–11)/11 = 445%) percentage change in *f*_150_ after virtual knockout of *L*-type calcium ion channels.

These examples emphasize the need to assess models employing unbiased stochastic searches, and the need to account for the heterogeneities inherent to neuronal populations. If a hand-tuned model, which let’s say arrives at parameters that are close to one of these four models, the conclusions would be based *solely* on the ion channel composition in that single hand-tuned model, arriving at biased conclusions about the role of specific ion channels in regulating specific measurements across the entire population of neurons. Thus, the heterogeneous population arrived employing the unbiased stochastic search and the VKM approach together enabled recognition and quantification of the differential and variable dependence of different measurements on distinct ion channels in disparate models. Specifically, the terms *differential* and *variable* are employed here to emphasize two distinct findings: (i) the effect of a single ion channel knockout is not the same across different physiological measurements studied, implying a *differential* impact across measuements; and (ii) the effect of knocking out a specific ion channel on a single physiological measurement is not the same, thus constituting a *variable* impact.

### 3.2 Differential impact of voltage-gated potassium and HCN ion channels on intrinsic properties of granule and basket cells

Of the two voltage-gated potassium ion channels incorporated into GC models, the absence of the non-inactivating KDR ion channels resulted in improper repolarization of action potentials, and did not considerably alter sub-threshold properties (*e.g*., Fig. 2). The lack of transient KA VKMs significantly altered all supra-threshold measurements (Fig. 3A–F), with greater impact on AP amplitude (Fig. 3A) and AP half-width (Fig. 3B), also manifesting significant variability across models (Fig. 3A–F). Importantly, although elimination of a potassium ion channel is generally expected to *increase* excitability, in a large proportion of models, we observed a counterintuitive *reduction* in firing rate after virtual knockout of KA ion channels (Fig. 3F). However, such counterintuitive changes to firing rates have been explained through *functional interactions* across ion channels (Kispersky et al., 2012), and the interactions between KA and other repolarizing channels have been shown to explain similar counter-intuitive results observed with changes in KA ion channel conductances (Anirudhan and Narayanan, 2015; Narayanan and Johnston, 2010).

In our case, the explanation emerged from the impact of KA ion channels on other measurements (Fig. 3 and Fig. S1). Specifically, in KA VKMs, the AP amplitude (Fig. 3A) and AP half width (Fig. 3B) are considerably larger, implying a higher degree of voltage-dependent activation of KDR ion channels (Fig. S1B) and a larger influx of calcium through the voltage-gated calcium ion channels (Fig. S1C), resulting from the large-amplitude and wide action potentials. This, in turn, results in a larger fraction of the other repolarizing ion channels (KDR, SK and BK) opening (Fig. S1D), reflected in a larger afterhyperpolarization (Fig. 3D, Fig. S1A) and a higher adaptation (Fig. 3E, Fig. S1A). The large afterhyperpolarization leads to a longer time for the membrane to charge up to threshold, and together with the higher adaptation resulted in the observed reduction in firing rates in the large proportion of models. Thus, the relative dominance of other ion channels in the repolarization kinetics and the enhanced action potential amplitude in the absence of KA ion channels explains the *counterintuitive* reduction in firing rate in their VKMs. As expected from the inactivating nature of these ion channels and the hyperpolarized resting potentials of GCs, KA ion channels did not significantly affect subthreshold measurements (Fig. 3G and H).

The lack of hyperpolarization-activated cyclic nucleotide gated ion channels (HCN or *h*) in GCs introduced large and variable changes to input resistance (Fig. 3G) and sag ratio (Fig. 3H), given its expression at rest as well as its hyperpolarization-induced activation profile. This robust impact on sub-threshold properties of neuron also translated to mild changes to other supra threshold measurements (Fig. 3A–F).

In the BC population, very similar to their counterparts in the GC population, HCN ion channels dominantly influenced sub-threshold measurements (Fig. 3G and H). However, in contrast to majority of the GC models, here VKMs of KA ion channels exhibited strong *increase* in firing rate (Fig. 3F), which was consistent with a lack of change in AP amplitude (Fig. 3A) and half width (Fig. 3B) in KA VKMs of these neurons.

### 3.3 Differential impact of voltage-gated calcium and calcium-activated potassium ion channels on intrinsic properties of granule cells

The granule cell model employed in this study expressed three voltage-gated calcium ion channels. We noted that the elimination of the non-inactivating CaL ion channels had the largest impact on ISI ratio (Fig. 3E) and firing rate (Fig. 3F), but showed very little effect on both subthreshold measurements (Fig. 3G–H). There was significant variability on how these ion channels altered firing rate and ISI ratio on different models (Fig. 3E and F). Elimination of the inactivating *N*-type calcium ion channels did not introduce large changes in any of the sub- or supra-threshold measurements considered here, and the variability across models was lower as compared to the variability across CaL VKMs. VKMs of the low-voltage activated inactivating CaT ion channels exhibited small and variable changes in AP threshold (Fig. 3C), fast afterhyperpolarization (Fig. 3D), ISI ratio (Fig. 3E) and firing rate (Fig. 3F). In addition, given their low-voltage activation, CaT ion channels also impacted input resistance to a small extent (Fig. 3G).

Turning to calcium-activated potassium ion channels, the VKMs of either BK or SK ion channels showed the largest and highly variable impact on ISI ratio (Fig. 3E) and on firing rate (Fig. 3F), having relatively weaker impact on the other sub and supra threshold measurements. Based on the similarity in the outcomes of knocking out CaL or the calcium-activated potassium ion channels on physiological responses, we reasoned the increase in excitability after CaL knockouts to be consequent to their interactions with the calcium-dependent potassium ion channels. Our BC model did not contain any calcium- or calcium-activated potassium channels.

Together, our analyses of the impact of different ion channels on single-neuron physiology of the heterogeneous GC and BC populations demonstrated differential and variable dependence of the various physiological measurements on these ion channels. Importantly, the mapping between ion channels and physiological measurements was many-to-many (but not all-to-all), where different ion channels affected any given measurement (*differential*) and any specific ion channel altered several measurements (*variable*).

### 3.4 Virtual knockout of individual ion channels across GCs introduced differential and variable scaling of firing rate profiles and associated spatial maps in DG network

**Fig. 6:**
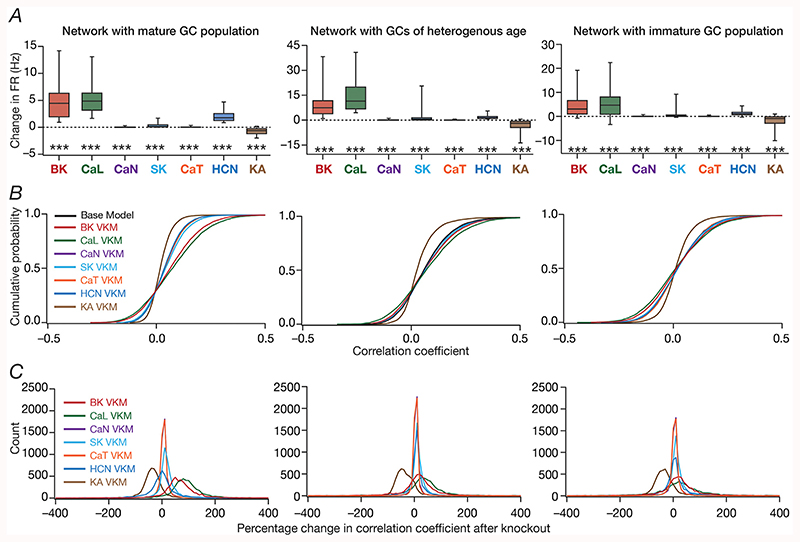
Virtual knockout of individual ion channels from granule cells resulted in differential and variable impact on channel decorrelation in networks endowed with distinct heterogeneities and receiving *heterogeneous* afferent inputs. *A*, Difference in firing rates (Eq. (12)) for all granule cells in the network, represented as quartiles. Firing rate of each cell was computed from the spike count of the cell for the entire 1000 s traversal of the virtual animal. *p* values were obtained using Wilcoxon signed rank test, where the change in firing rate was tested for significance from a “no change” scenario. ***: *p* < 0.001. *B*, Cumulative distribution of inter-neuronal pairwise firing rate correlation coefficients for networks built with either the base models, or with models after virtual knockout of individual ion channels. Shown are plots for the base model network and for the networks built with GC neurons where one of the 7 ion channels was virtually knocked out. In comparing the graphs in panel *B* to those in Fig. 5B, note that the *X* axes of graphs in this panel span -0.5 to 0.5, and not -1 to 1 as in Fig. 5B. *C*, Distribution of percentage changes in correlation coefficients for neuronal responses from the VKM network, compared to the respective base model coefficients. Shown are plots corresponding to percentage changes in networks built with GC neurons where one of the 7 ion channels was virtually knocked out. For (*A*-*C*), plots are shown for simulations performed with three distinct networks and associated virtual knockouts: network with a fully mature GC population (left), network with a GC population of heterogeneous age (*center*) and network with a fully immature GC population (*right*). Note that all three networks are endowed with intrinsic and synaptic heterogeneities. Neurons in the network received heterogeneous afferent inputs.

We built a model of the DG microcircuit comprised of GCs and BCs, receiving local-circuit connections and afferent inputs that were driven by the movement of virtual animal, to assess the impact of ion-channel elimination on network function. We computed the firing rate profile and associated spatial maps of individual GCs for the entire arena (Fig. 1C), and assessed the impact of individual ion-channel knockouts from GCs on their firing rate profiles and spatial maps with reference to the virtual animal traversal (Fig. 1C). In performing VKM simulations at the network scale, everything else in the network was set identical to baseline conditions, including the specific spatio-temporal trajectory of the virtual animal in the arena, except for knocking out one specific ion channel from all GCs in the network. The procedure was repeated for the 7 GC ion channels, and the firing rates of networks built with the VKMs were compared with those of the network with base models, under two scenarios involving neurons in the network receiving identical (Figs. 4 and 5) or heterogeneous (Fig. S2, Fig. 6) afferent inputs.

The impact of virtually knocking out each of the 7 GC ion channels on firing rate profiles and spatial map profiles of four different example GCs residing in two distinct networks are shown in Fig. 4 (*identical* afferent inputs) and Fig. S2 (heterogeneous afferent inputs). Reminiscent of observations with single-neuron physiological measurements (Fig. 3), we found *differential* impact of different ion channel knockouts on the profiles of the same neuron, and *variable* impact of knocking out the same ion channel on different neurons within the same network. For instance, in Fig. 4, in GC #50 (Fig. 4A), deletion of either HCN or BK ion channels resulted in large increases in firing rate, whereas KA VKMs exhibited reductions in firing rates and the other knockouts did not elicit significant differences in firing rate profiles across space. With reference to variability in the impact of knockouts, in GC #44 within the same network (Fig. 4B) recorded during the same virtual traversal, BK, CaL or CaT VKMs did not have a significant effect, but deletion of CaL, SK or HCN ion channels resulted in increased firing rates and KA VKMs again exhibited reduced firing rates. The firing rate changes were introduced by knockouts belonged to two broad categories: multiplicative scaling, where changes were restricted to the locations where the base model fired (*e.g*., SK VKM of GC #44 compared to its baseline of Fig. 4B), and additive scaling, where there was a shift in the entire firing profile (*e.g*., HCN VKM of GC #44 compared to its baseline of Fig. 4B). In some cases, we observed a combination of multiplicative and additive scaling (*e.g*., BK VKM of GC #50 compared to its baseline of Fig. 4A). As the network depicted in Fig. 4 received *identical* EC inputs, it may be noted that the place field locations were identical across the two cells, with differences only in firing rates between these two cells and across knockouts. We confirmed these observations to also extend to a network endowed with afferent heterogeneities (Fig. S2). Specifically, the differential (*e.g*., SK vs. BK VKMs of GC #84 in Fig. S2) and variable (*e.g*., BK VKMs of GC #84 in Fig. S2A *vs*. GC #44 in Fig. S2B) responses to ion channel knockouts in the same set of neurons within the same network during the same virtual traversal were observed. Firing rate changes were scaled either multiplicatively (*e.g*., KA VKMs of GC #44 in Fig. S2B) or additively (*e.g*., HCN VKMs of GC #44 in Fig. S2B) or both (*e.g*., BK VKMs of GC #44 in Fig. S2A). However, as the network depicted in Fig. S2 received heterogeneous EC inputs, the place field locations were different between the two cells within the same network during the same virtual traversal. Virtual knockouts, however, merely scaled the firing rates of individual cells, without altering the position of place fields where a given cell was firing (*e.g*., compare the firing profiles and spatial maps of GC #84 in Fig. S2A under baseline conditions and in the VKM network).

### 3.5 Virtual knockout of individual ion channels across GCs resulted in larger changes in firing rate profiles in networks containing immature neurons

We quantified these variable and differential responses of neural firing to the virtual knockout of different ion channels across the entire population of GCs for network receiving *identical* (Fig. 5A) or heterogeneous (Fig. 6A) EC inputs, with different degrees of neurogenesis-driven structural heterogeneity. With reference to degrees of structural heterogeneities, we employed three configurations (Mishra and Narayanan, 2019): two networks with fully mature or fully immature GC populations, which did not have any structural heterogeneities and a network that was endowed with a heterogeneous structural properties (Figs. 5 and 6). Whereas the fully mature population refers to a scenario where there are no new neurons integrated into the circuit, the fully immature population is an artificial setting where all neurons are immature with less surface area and the heterogeneous population is reflective of a more natural milieu where neurons are at different stage of maturation. Although the quantitative changes in firing rate were dependent also on the specific kind of structural heterogeneities expressed in the network, the direction and strength of changes in GC firing rate in a network were similar to those at the single-neuron scale (compare Figs. 5A and 6A with Fig. 3F). Specifically, in a large proportion of GCs, knockout of CaL or BK ion channels introduced large increases in firing rate, elimination of CaN or CaT or HCN or SK ion channels resulted in relatively smaller increases in firing rate, and KA ion channel VKMs exhibited relatively small reduction in firing rate compared to their respective base models. Interpretations of changes, however, should not be drawn from the summary statistics, but should be driven by heterogeneities (Rathour and Narayanan, 2019; Marder and Taylor, 2011). It should be noted that there are significant differences in different neurons in the same network during the same virtual traversal in terms of which ion channel plays a dominant role in altering firing rate (Figs. 4–6; Fig. S2).

Quantitatively, although the strength of afferent drive was scaled depending on the maturity (surface area) of the neuron to ensure that network firing rates were comparable across the three networks, VKMs resulted in larger changes in firing rates in networks endowed with immature neurons. This was irrespective of whether the network received *identical* (Fig. 5A) or heterogeneous (Fig. 6A) afferent inputs. This should be expected because of the higher excitability of the relatively immature neurons, whereby even smaller changes to currents (here due to loss of specific ion channels) result in larger changes to voltage responses. Together, virtual knockout of individual ion channels across GCs resulted in differential and variable scaling of firing rate profiles and associated spatial maps, with larger changes observed in networks where immature neurons were present.

### 3.6 The impact of eliminating individual ion channels on channel decorrelation was differential and variable in networks endowed with different heterogeneities

The anatomical location of the DG, its unique features of sparse and diverse connectivity in conjunction with the expression of adult neurogenesis has led to postulates of its role in response decorrelation and pattern separation. Channel decorrelation, one form of decorrelation of network responses, is assessed by computing pair-wise correlations across temporally aligned outputs of individual neurons (information channels) within the network, when inputs corresponding to a single virtual arena traversal arrive onto the network. It has been established that distinct forms of network heterogeneities could synergistically interact with each other in mediating channel decorrelation in the DG, apart from establishing a dominance hierarchy among these forms of heterogeneities when they co-expressed (Mishra and Narayanan, 2019). Does altering neuronal intrinsic properties through virtual knockout of individual ion channels regulate channel decorrelation in the DG network?

We plotted the distribution of pairwise correlation coefficients of firing rate profiles of the different GCs in the network, for the base network (where all ion channels were intact), and for each of the 7 GC VKMs. We repeated this procedure for scenarios where inputs from EC were *identical* (Fig. 5B) or heterogeneous (Fig. 6B), for different degrees of structural heterogeneity in each case. We found differential effects of knocking out different ion channels on channel decorrelation in these networks. Consistent with previous observations (Mishra and Narayanan, 2019), we noted that the degree of decorrelation observed in networks with heterogeneous afferent inputs (Fig. 6B) was higher than networks with *identical* afferent inputs (Fig. 5B). We also confirmed prior observations (Mishra and Narayanan, 2019) that the impact of different structural heterogeneities on networks receiving *identical* EC inputs was higher than the impact on networks receiving heterogeneous EC inputs (compare the base model distributions of correlation coefficients across the three different forms of heterogeneities in Fig. 5B vs. Fig. 6B). However, qualitatively, the effects of knocking out different ion channels on channel decorrelation were consistent across different network configurations, each endowed with distinct afferent and structural heterogeneities (Figs. 5B and 6B).

Specifically, across the different network configurations, elimination of either BK or CaL ion channels from the GCs of the network resulted in large rightward shifts in the cumulative distribution of response correlation coefficients, indicative of a reduction in channel decorrelation (Figs. 5B and 6B). On the other hand, virtual knockout of KA ion channels resulted in a significant leftward shift in the cumulative distribution of response correlation coefficients, indicating enhanced decorrelation in these networks. The other VKMs, of CaN, CaT, SK and HCN ion channels, resulted in relatively smaller shifts to the correlation coefficient distributions (Figs. 5B and 6B). These observations were further confirmed by the distributions of VKM-induced percentage changes in correlation coefficients for networks receiving *identical* (Fig. 5C) or heterogeneous (Fig. 6C) afferent inputs, and endowed with different degrees of structural heterogeneities.

Together, these analyses demonstrated that the impact of eliminating individual ion channels from DG granule cells on network-scale channel decorrelation was differential and variable, in heterogeneous networks receiving either identical or heterogeneous afferent inputs.

### 3.7 Networks endowed with high-excitability immature neurons manifested resilient channel decorrelation after virtual knockout of ion channels

The correlation plots presented with scenarios where the DG network received identical (Fig. 5) or heterogeneous (Fig. 6) afferent inputs were with reference to the response correlation of the network output. Whereas such macroscopic analyses of network outcome provides insights about the overall ability of the network to discriminate, it is essential that output correlations are assessed with reference to their respective input correlations, spanning all pairs of neurons in the network. Across pairs of neurons in the network, how did the elimination of individual ion channels alter output response correlation as a function of different input correlations? Did ion channel knockouts specifically affect inputs with lower or higher correlation? Did the presence of neurogenesis-induced structural differences in the DG network alter the quantitative impact of different ion channel knockouts on channel decorrelation?

**Fig. 7:**
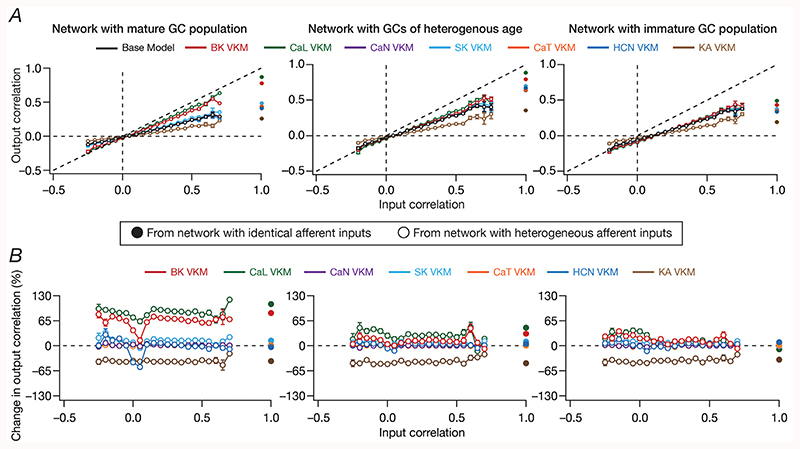
The impact of virtual knockout of individual ion channels on the degree of channel decorrelation depends on the specific ion channel being knocked out, and reduced in the presence of immature neurons. *A*, Pairwise response (output) correlation plotted as a function of the corresponding pairwise input correlation, for the base model network and for networks built with GC neurons where one of the 7 ion channels was virtually knocked out. *B*, Percentage change in response (output) decorrelation in VKM networks with reference to the corresponding channel decorrelation in the base model, plotted as functions of input correlation. For (A-B), plots are shown for simulations performed with three distinct networks and associated virtual knockouts: network with a fully mature GC population (*left*), network with a GC population of heterogeneous age (*center*) and network with a fully immature GC population (*right*). Note that all three networks are endowed with intrinsic and synaptic heterogeneities. In all cases, network outcomes are represented as solid or open circles, when the network received identical or heterogeneous afferent input, respectively. Note that the input correlation is unity for networks receiving identical inputs, whereas input correlation is dependent on specific pairs of inputs when the network receives heterogeneous inputs.

To address these questions, we placed output correlation coefficients of neuronal pairs into specific bins corresponding to their respective input correlation coefficients, and plotted the statistics of output correlations as functions of input correlations (Mishra and Narayanan, 2019). Our experimental design involving identical and heterogeneous afferent inputs provided us an ideal setting to assess output correlations over a broad span of input correlations. Specifically, whereas the network with *identical* afferent inputs had an input correlation of unity across all neuronal pairs, the network with heterogeneous afferent inputs was endowed with a range of pairwise input correlation coefficients depending on the specific nature of inputs that the neurons received (Fig. 7A). As established earlier (Mishra and Narayanan, 2019), the base network (where all ion channels were intact) manifested robust channel decorrelation, whereby the average output correlation coefficients were lesser than the average input correlation across all observed input correlations (Fig. 7A; black traces).

When we measured the output correlations as functions of their respective input correlations after elimination of individual GC ion channels, we made two important observations. First, in the presence of immature neurons, either in a network that was constructed entirely with immature neurons or in a network that was endowed with structural heterogeneities, we found that the amount of VKM-induced changes in channel decorrelation to be lesser compared to a network that was constructed of mature neurons (Fig. 7A). This was also reflected in the percentage changes observed in output correlation plotted as functions of input correlations (Fig. 7B). Second, for a given VKM, the average percentage change in output correlation was invariant to the average input correlation, across networks endowed with different structural and afferent heterogeneities (Fig. 7B). This implies that under molecular perturbations, in spite of the dominant nature of afferent heterogeneity (Mishra and Narayanan, 2019) over local heterogeneities in mediating channel decorrelation, elimination of different ion channels results in differential impacts on response decorrelation. Although some VKMs elicited an enhancement and other introduced a reduction in the correlation coefficients, and although the quantitative changes were dependent on the specific heterogeneities expressed in the network (Figs. 5–7, Fig. S2), the average percentage changes in output correlation was largely independent of the specific values of input correlation (Fig. 7B). This observation extended to output correlation coefficients measured for *identical* inputs (input correlation coefficient = 1) as well (Fig. 7B).

**Fig. 8:**
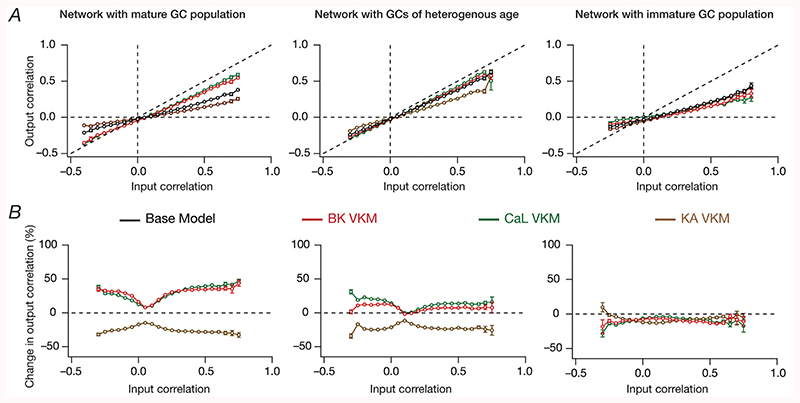
Functional resilience to ion-channel elimination introduced by the incorporation of immature neurons was prevalent in a larger network. *A*, Pairwise response (output) correlation plotted as a function of the corresponding pairwise input correlation, for the base model network and for networks built with GC neurons where 3 of the 7 ion channels was virtually knocked out. *B*, Percentage change in response (output) decorrelation in VKM networks with reference to the corresponding channel decorrelation in the base model, plotted as functions of input correlation. For (*A-B*), plots are shown for simulations performed with three distinct networks and associated virtual knockouts: network with a fully mature GC population (left), network with GC population of heterogeneous age (middle) and network with a fully immature GC population (right). Note that all the networks are endowed with intrinsic and synaptic heterogeneities.

To test the robustness of our conclusions to changes in network size, we repeated our analyses in Fig. 7 with a larger network size comprised of 500 GCs and 75 BCs (Fig. 8). Based on the above results on ion channels whose knockout results in significant and large changes to channel decorrelation, we selectively performed virtual knockout simulations for three ion channels: BK, CaL and KA. Given the comparatively large computational cost associated with such large conductance-based networks, we performed the simulations for two network configurations that were endowed with intrinsic and synaptic heterogeneities, but differed only in terms of structural heterogeneity: a network comprised of mature cells (Fig. 8A, *left*), other comprised of immature DG neurons (Fig. 8A, *right*) and a third that was endowed with neurogenesis-induced structural heterogeneities (Fig. 8A, *middle*). Consistent with our conclusions with a smaller network, we found that networks comprised of immature neurons were resilient to the ion channel perturbations as compared to the network endowed with only intrinsic and synaptic heterogeneity (Fig. 8).

Together, these analyses demonstrated that the impact of ion channel elimination on channel decorrelation was lower in the presence of structurally immature high-excitability neurons. In addition, these analyses show that for any given ion channel knockout, the average *percentage change* in output correlation was invariant to the specific values of input correlation. Importantly these conclusions on functional resilience in the presence of structurally immature neurons and on the invariance of percentage changes in output correlation to specific values of input correlations were robustly observed in networks of different sizes.

### 3.8 Virtual knockout of either the KA or the HCN ion channels from basket cells did not significantly alter GC firing rates or network decorrelation

Thus far, our analyses were confined to VKMs of GC neurons. What is the impact of altering ion channel composition in the basket cells of the network? We measured GC firing rates and decorrelation across GC responses in networks built with BCs lacking either the KA or the HCN ion channels (Fig. S3). We compared these outcomes with the base network (where all ion channels were intact), and found that elimination of either KA or HCN ion channels from BCs did not considerably alter GC firing rates across the network or introduce prominent changes in channel decorrelation computed across GC responses. This was consistent across networks with different configurations involving disparate combinations of structural and afferent heterogeneities (Fig. S3). We noted these to be simply a reflection of the relatively minor role played by the two ion channels in regulating BC intrinsic properties (Fig. 3).

## 4 Discussion

In this study, employing sequential multi-scale analyses, we systematically assessed the impacts of eliminating individual ion channels on single-neuron physiological properties, on network excitability and on channel decorrelation in DG networks. At the single-neuron scale, our analyses revealed that the mapping between ion channels and physiological measurements was many-to-many. At the network scale, the impact of knocking out individual ion channels was differential and variable both in terms of affecting network firing rates and channel decorrelation, but also was critically reliant on the specific local heterogeneities expressed in the DG network. Importantly, in the presence of structurally immature neurons in the DG network, the impact of ion channel elimination on channel decorrelation was considerably lower when compared with a network exclusively constructed with structurally mature neurons. These results highlight the role of local heterogeneities in regulating the resilience of the DG network to large network-wide perturbations to neuronal ion channel composition. Our analyses also showed that for a given VKM, the average percentage change in output correlation was invariant to the specific values of input correlation.

### 4.1 Heterogeneities in ion channel regulation of neuronal and network physiology

The ubiquitously expressed variability in ion channel expression in each of the several neuronal subtypes imparts unique features to single neuron and network physiology. First, this variability forms the substrate for ion-channel degeneracy where similar physiological outcomes are achieved through disparate combinations of ion channels and their properties. This provides neurons with considerable flexibility in maintaining robustness of their signature physiological characteristics, without strong constraints on ion channel expression profiles (Drion et al., 2015; Rathour and Narayanan, 2019; Goldman et al., 2001; Marder and Goaillard, 2006). Second, the variability in ion channel expression profiles implies that the response of neurons to even *identical* stimuli could be distinct, depending on the specific ion channels that are expressed, on the state of the neuron, on the afferent stimulus and how they activate/deactivate/inactivate the different ion channels, on the impact of different neuromodulators, and on activity-dependent plasticity profiles of and interactions among these ion channels. This allows such intrinsic variability to form *a* substrate for decorrelating afferent stimuli (Mishra and Narayanan, 2019; Padmanabhan and Urban, 2010).

Third, depending on variable expression and the specific interactions among different ion channels, emergent properties could result in counter-intuitive observations that are perfectly explained by synergistic interactions (Drion et al., 2015; Kispersky et al., 2012). For instance, an increase in KA conductance is typically expected to reduce excitability, consequently reduce the calcium influx into the cytosol and shift frequency-dependent plasticity profiles to the right. However, under certain scenarios, owing to interactions of these conductances with the KDR conductance, an increase in KA conductance results in enhanced calcium influx and leftward shifts to plasticity profiles (Anirudhan and Narayanan, 2015; Narayanan and Johnston, 2010). Similarly, we observed a counter-intuitive reduction in firing rate as a consequence of eliminating KA conductances. This was effectuated by the enhanced activation of other potassium ion channels consequent to larger and wider action potentials (Fig. 3).

Although the expression of KA conductances in DG granule cells has been established (Beck et al., 1992; Peng et al., 2013; Alfaro-Ruiz et al., 2019; Serodio and Rudy, 1998; Sheng et al., 1992; Varga et al., 2000; Rhodes et al., 2004; Monaghan et al., 2008; Ruschenschmidt et al., 2006), heterogeneities in the impact of acutely eliminating KA ion channels have not been systematically assessed. Future studies should employ different techniques to assess heterogeneities in the impact of such elimination on neuronal firing rates. These experiments are especially important in light of the strong expression of calcium-activated potassium ion channels in DG granule cells (Aradi and Holmes, 1999; Mateos-Aparicio et al., 2014; Brenner et al., 2005; Sailer et al., 2002). These acute blockade experiments should be coupled with systematic location-dependent recordings of KA currents (Hoffman et al., 1997) from the dendrites of DG granule cells, coupled with morphologically realistic computational models accounting for subcellular ion channel distributions (Beining et al., 2017). Such experiments would provide important insights about the specific roles of gradients in ion channel expression and spatiotemporal interactions between ion channels (Rathour et al., 2016; Rathour and Narayanan, 2012a; Rathour and Narayanan, 2012b, 2014).

In relation to the impact of neural heterogeneities mentioned above, our previous study (Mishra and Narayanan, 2019) established degeneracy in the emergence of channel decorrelation, specifically demonstrating that disparate forms of heterogeneities could combine to elicit similar levels of channel decorrelation. We had demonstrated that local heterogeneities contribute to decorrelation of identical afferent stimuli, and had established a dominance hierarchy among different forms of heterogeneities, specifically showing afferent heterogeneities to be the dominant form (Mishra and Narayanan, 2019). In contrast, here our focus is on assessing the specific roles of individual ion channels in channel decorrelation. In addition, we demonstrate a critical role for distinct forms of *local* heterogeneities, specifically of neurogenesis-induced structural heterogeneities, in providing functional resilience in the face of perturbations. We have made explicit testable predictions about individual ion channels, and explore in detail the mechanistic basis for why results were the way they were (including counterintuitive conclusions such as the ones involved with *A*-type potassium ion channel knockout).

From the spatial encoding perspective, hippocampal place maps are known to be flexible, whereby the neural code of space remaps to mirror the animal’s behavioral experience (Dupret et al., 2010). One such remapping is where the firing rate of a place cell could undergo changes in response to environmental changes, with these changes also changing in a field-specific manner (Leutgeb et al., 2005, 2007). Such rate remapping has been postulated to permit the distinctiveness of sensory events while maintaining the integrity of the spatial code (Renno-Costa et al., 2010). Our results show that modulation of intrinsic properties, either through neuromodulatory action or through activity-dependent plasticity could form a putative substrate for rate remapping. Within this framework, field-specific rate remapping (Leutgeb et al., 2005, 2007) could be achieved through differential neuromodulatory tones that are altered in a field-specific manner (as a potential consequence of behavioral associations to individual fields).

Together, we propose that the multi-scale approach presented and analyzed here, involving multiple forms of biological heterogeneities could be employed as a powerful tool to assess the cascading impact of *lower-scale perturbations* to *higher-scale function*. Although our analyses has been focused on knockouts of individual ion channels, this approach could be extended to the analyses of graded perturbations to ion channels and to other molecular components, including receptors, pumps, transporters and signaling molecules involved in regulating cellular physiology.

### 4.2 Variability breeds robustness: Implications for the expression of local heterogeneities on ion channel regulation of channel decorrelation

Although there are several detailed studies on the variable role of individual ion channels in altering single neuron physiology (previous section), the extension of such analyses assessing the variable roles of individual ion channels to network scale functions have been far and few (Padmanabhan & Urban, 2010, 2014; Prinz et al., 2004; Morgan et al., 2007; Schneider et al., 2012; Yim et al., 2015). Our analyses highlight the importance of individual ion channels and local heterogeneities to network-scale decorrelation *even* with the expression of the dominant afferent heterogeneities.

From a memory-encoding standpoint, it has been argued that DG neurons could act as engram cells, through changes in neuronal properties including cell-autonomous plasticity to membrane excitability (Titley et al., 2017; Tonegawa et al., 2018; Zhang and Linden, 2003; Yim et al., 2015; Stegen et al., 2012; Rao-Ruiz et al., 2019; Josselyn and Frankland, 2018; Kim and Linden, 2007; Gallistel, 2017). How do such changes in intrinsic properties alter network-scale decorrelation? From a pathophysiological standpoint, acquired or inherited channelopathies are associated with several neurological disorders and have been shown to be prevalent within the DG as well (Brenner et al., 2005; Kirchheim et al., 2013; Young et al., 2009; Stegen et al., 2009; Bender et al., 2003; Surges et al., 2012; Kohling and Wolfart, 2016; Beck and Yaari, 2008). How does the network respond to such strong perturbations to ion channel composition, especially from the functional standpoint of response decorrelation? In addressing these questions, our analyses show that mnemonic or pathophysiological intrinsic plasticity could alter the degree of decorrelation, apart from providing a quantitative framework to address this question in a neuron-specific and ion channel-specific manner. We postulate that the expression of local heterogeneities could help the network stay resilient to large ion channel perturbations. The specific impact of these ion-channel perturbations to channel decorrelation would depend on several factors, including the identity of the ion channel(s) involved, the answer to the question on what *other* component(s) changed, the nature of inputs to the network, the interactions of these altered components with other components in the network and the specific sets of heterogeneities expressed in the network.

Furthermore, during the maturation process following generation of new neurons, it has been established that certain ion channels might not express during early stages of maturation (Lodge and Bischofberger, 2019; Ambrogini et al., 2004; Overstreet-Wadiche et al., 2006; Overstreet-Wadiche and Westbrook, 2006; Piatti et al., 2006). Therefore, the scenario analyzed here with the elimination of specific ion channels in immature neurons is equivalent to the absence of these ion channels during maturation.

### 4.3 Limitations and future directions

In our study, we had analyzed the impact of complete elimination of individual ion channels. However, our conductance-based multi-scale modeling framework could be employed to assess the impact of perturbation to specific sets of ion channels that are observed under physiological or pathophysiological conditions. In such scenarios, the heterogeneous impact of changes could also be incorporated within the framework to make specific predictions on how network function would change under heterogeneous plasticity in different network components. Although our analyses here are with reference to channel decorrelation, future studies could explore the impact of individual ion channels on pattern decorrelation within the same framework, which would provide specific insights into the role of distinct forms of heterogeneities and different ion channels on pattern separation. Such analyses should focus on ion-channel regulation of separation of input patterns, encoded not just as firing rate but also through temporal codes (Madar et al., 2019).

In this study, our focus was on perturbations to ion channels expressed in the GC and BC populations. The network model did not incorporate other DG cell types, including the mossy cells, the molecular layer perforant path-associated cells, the semilunar granule cells and other interneurons that are prevalent within the DG (Amaral et al., 2007; Li et al., 2012; Scharfman and Myers, 2012; Williams et al., 2007). Future studies could explore the impact of heterogeneities and ion-channel perturbations in these different neuronal subtypes on neuronal network physiology. Furthermore, we incorporated adult neurogenesis into the DG network through three changes: (i) structural changes in neurons reflecting reduced surface area of granule cells thereby matching the increased excitability of immature cells (Aimone et al., 2014; van Praag et al., 2002); (ii) reduction of the overall afferent drive to neurons based on their surface area, so that reduced drive in immature neurons counterbalanced their high excitability Li et al. (2017); Mongiat et al. (2009); Dieni et al. (2016); and (iii) the orthogonal afferent connectivity, actively driven by adult neurogenesis (Li et al., 2017; Lodge and Bischofberger, 2019; Aimone et al., 2006, 2009; Luna et al., 2019), was incorporated as afferent heterogeneities into the network model. Future models could incorporate the array of neurogenesis-induced changes, including those in the expression of ion channels, receptors, calcium handling and differential plasticity profiles (Li et al., 2017; Lodge and Bischofberger, 2019; Aimone et al., 2006, 2009, 2014; Luna et al., 2019; van Praag et al., 2002; Mongiat et al., 2009; Gonzalez et al., 2018; Stocca et al., 2008), into heterogeneous network models that also account for activity-driven plasticity and the emergence of afferent heterogeneities.

The testable predictions presented here on the specific roles of individual ion channels could be electrophysiologically tested, both from the single-cell and network perspectives employing pharmacological agents to block specific ion channels or employing genetic methods to silence specific ion channels. Our analyses also presents a specific testable prediction on the role of neurogenesis-induced heterogeneities in confering functional resilience of the DG network to molecular-scale perturbation. These predictions could be directly tested by recording spike trains from multiple DG granule cells as the animal traverses an arena in the presence of ion channel blockers or manipulations that would alter adult neurogenesis, and computing channel decorrelation of firing rates across these different neurons. In such analyses, interpretations should account for potential compensatory and activity-dependent plasticity mechanisms that follow the elimination of individual ion channels, and the possibility of *conjunctive* changes in *several* ion channels induced by activitydependent plasticity or neuromodulation or pathological conditions (Yim et al., 2015; Stegen et al., 2009, 2012; Young et al., 2009; Bender et al., 2003; Surges et al., 2012; Beck and Yaari, 2008; Mishra and Narayanan, 2020a). In the context of compensatory mechanisms triggered by ion-channel elimination, a limitation of our model is that analyses is restricted to the *acute* impact of ion channel elimination. Future computational studies could incorporate frameworks that account for compensations triggered by ion-channel knockouts through conjunctive changes in several channels (Srikanth and Narayanan, 2015; O’Leary, 2018; O’Leary et al., 2013; O’Leary et al., 2014) in analyzing the role of compensations in excitability and DG decorrelation.

## Supplementary Material

Figures S1–S3; Tables S1–S2

## Figures and Tables

**Table 1:** Experimentally derived electrophysiological measurements, lower and upper bounds that were employed for validating the granule cell and the basket cell models (Mishra and Narayanan, 2019). Data from (Aradi and Holmes, 1999; Krueppel et al., 2011; Lubke et al., 1998; Mott et al., 1997; Santhakumar et al., 2005).

	Measurement, Unit	Symbol	Granule cell		Basket cell	
			Lower	Upper	Lower	Upper
1	Action potential amplitude, mV	*V*_AP_	95	115	110	120
2	Action potential threshold, mV	*V*_th_	–55	–40	–51	–41
3	Action potential half-width, ms	*T*_APHW_	0.53	1.6	0.53	1.5
4	Fast after hyperpolarization, mV	*V*_fAHP_	–25	–3.4	–27	– 14
5	Sag ratio	Sag ratio	0.9	1	0.9	1
6	Spike frequency adaptation	SFA	0.1	0.8	0.9	1.04
7	Input resistance, MΩ	*R*_in_	107	228	45	65
8	Firing frequency at 50 pA, Hz	*f*_50_	0	0	0	0
9	Firing frequency at 150 pA, Hz	*f*_150_	10	15	30	50
