# Supplementary material for: Ion-channel regulation of response decorrelation in a heterogeneous multi-scale model of the dentate gyrus
Source: Curr Res Neurobiol. 2021 Mar 5;2:100007. doi: 10.1016/j.crneur.2021.100007 (PMC7610774; doi:10.1016/j.crneur.2021.100007)
Supplement: Multimedia component 1 [file mmc1.pdf]

Ion-channel regulation of response decorrelation in a heterogeneous multi-scale model of the dentate gyrus

Poonam Mishra and Rishikesh Narayanan*

Cellular Neurophysiology Laboratory, Molecular Biophysics Unit, Indian Institute of Science, Bangalore 560012, India.

Supplementary Material

Table of contents

Supplementary Figure S1 2
Supplementary Figure S2 3
Supplementary Figure S3 4

Supplementary Table S1 5
Supplementary Table S2 6

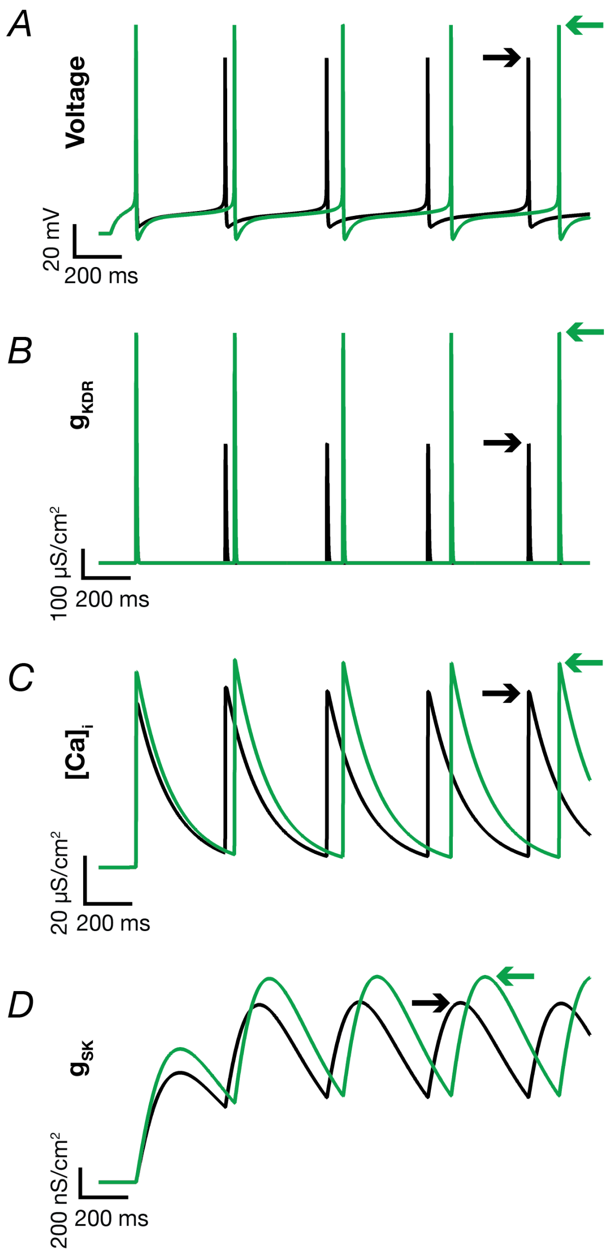

Figure S1. **Schematic representing counter-intutive reduction in firing rate of neuron in case of *A*-type potassium (KA) channel knockout**. *A*–*D*, Voltage response of an example neuron to a step-current injection (*A*), delayed rectifier potassium channel conductance (*B*), intracellular calcium concentration (*C*) and conductance of calcium- dependent small conductance potassium channel (*D*), in control case (black) and after KA knockout (green). Please note that, across panels, the green arrow representing KA knockout condition is higher than the black arrow representing control case. Please also note that the firing rate of the neuron decreases after KA knockout (panel A; note that the interspike intervals are higher in the green trace). These results were consistently observed across other model GCs as well.

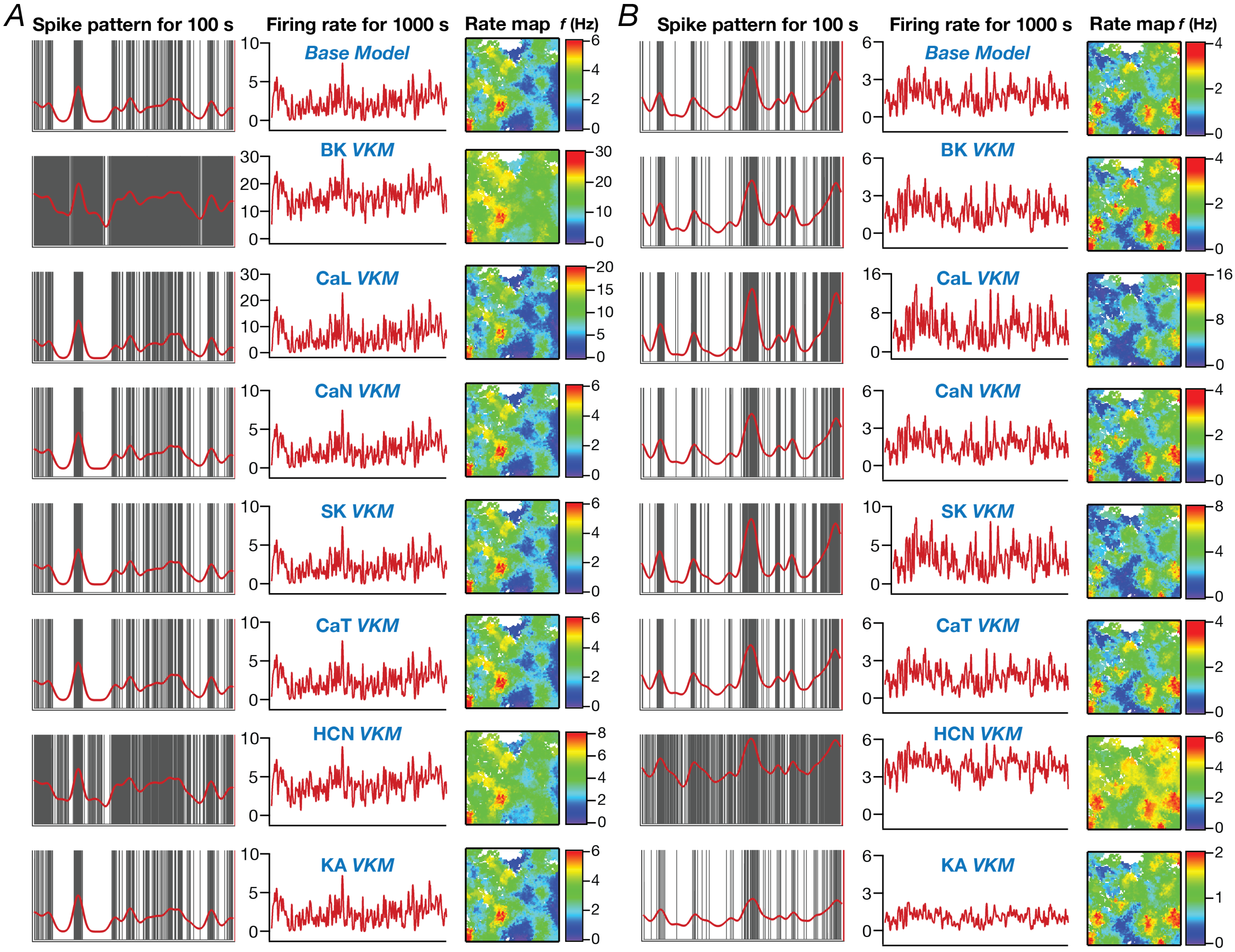

Supplementary Figure S2. Granule cell firing profiles and spatial maps depicting the heterogenous impact of virtually knocking out individual ion channels from granule cells in a network receiving *heterogeneous* afferent inputs. *A*, *Left:* Spike patterns (gray) overlaid with firing rates (red) for a 100 s period for valid GC model 84, residing in a GC-BC network endowed with intrinsic and synaptic heterogeneities and receiving identical afferent inputs. *Center:* Instantaneous firing rates of GC model 84 for the entire 1000 s of animal traversal across the arena. *Right:* Color-coded spatial rate maps showing firing rate of GC model 84 superimposed on the trajectory of the virtual animal. The top-most panels represents these measurements for the base model (where all ion channels are intact), and the other panels depict these measurements obtained after virtual knockout of individual ion channels from the granule cell population of the network. *B,* Same as (*A*) for GC model 44 residing in the same network. Models 84 and 44 respectively showed maximum and minimum changes in firing rate after virtual knockout of BK ion channel (see Fig. 5*A*). The network employed in this illustrative example was endowed with intrinsic and synaptic heterogeneities, but did not express structural heterogeneities.

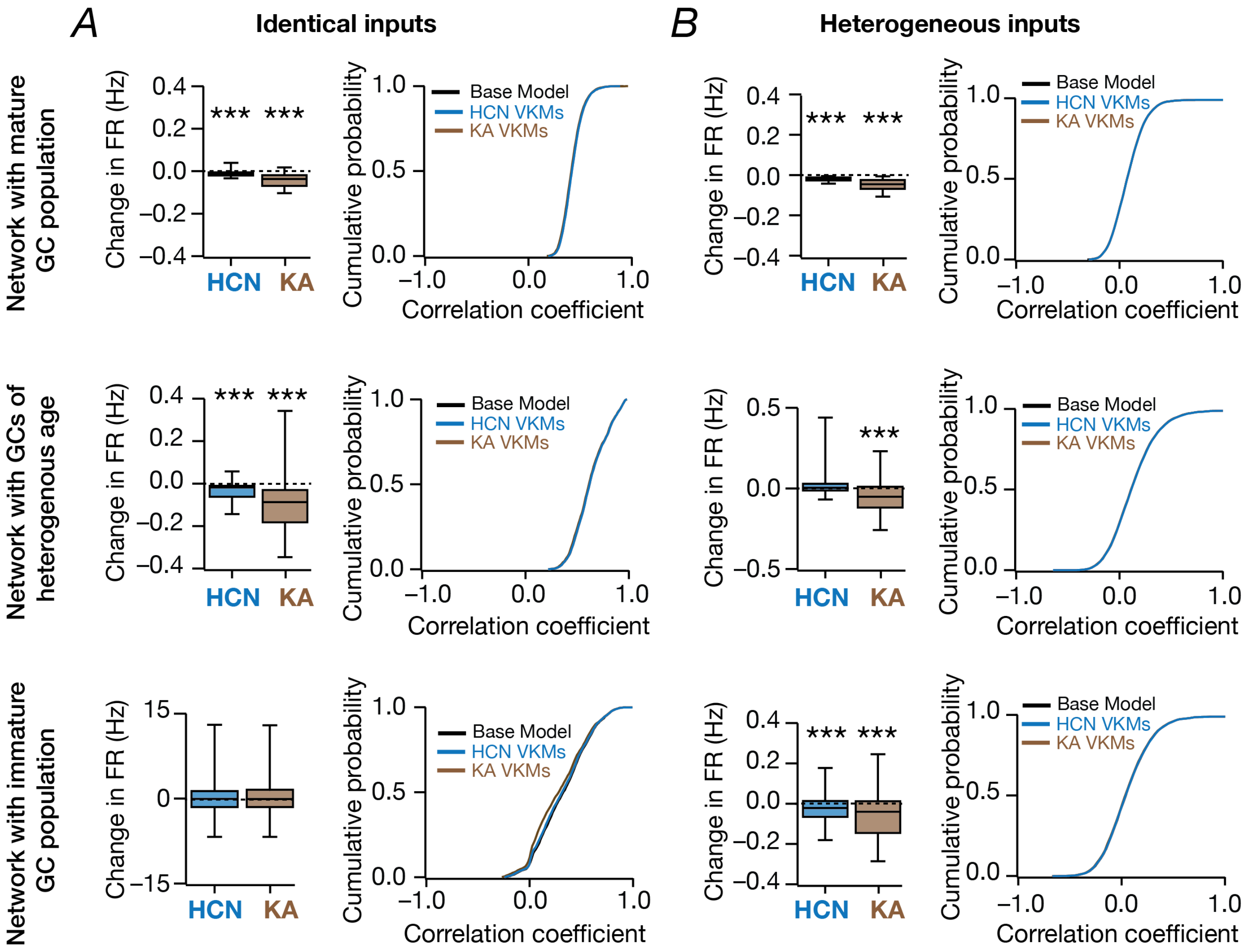

Supplementary Figure S3. **Weak impact of virtually knocking out HCN or KA ion channels from the basket cell population on firing rate and channel decorrelation in granule cell responses**. *A*, *Top*: *Left,* Change in granule cell firing rate in a network receiving *identical* afferent input, represented as quartiles. Shown are the cases for the HCN or KA ion channel knockout from the basket cell population. *Right,* Cumulative distribution of granule cell firing rate correlation coefficient for different ion channel knockouts. Shown are the cases for the baseline network, and for cases where HCN or KA ion channels were virtually knocked out from the basket cell population. Plots in the other panels are similar to the top panel graphs, but for networks with different age heterogeneities: network with a fully mature GC population (*top*), network with a GC population of heterogeneous age (*middle*) and network with a fully immature GC population (*bottom*). Note that all three networks are endowed with intrinsic and synaptic heterogeneities. *B,* Same as (*A*) for network receiving heterogeneous afferent inputs. *p* values were obtained using Wilcoxon signed rank test, where the change in firing rate was tested for significance from a “no change” scenario. *** $p < 0.001$.

Supplementary Table S1. Parameters and their ranges for stochastic search of valid granule cells (Mishra and Narayanan, 2019)

	Parameter	Symbol	Default	Testing Range
***h* channel properties**				
1	Maximal conductance (μS/cm^2)	h-g	5	2 to 12
2	Activation time constant of I_h (ms)	h-τ_A	39	30 to 50
3	$V_{1/2}$ activation of I_h (mV)	h-V_A	−81	−70 to −90
***A*-type K$^+$ channel properties**				
4	Maximal conductance (mS/cm^2)	KA-g	87	70 to 110
5	Activation time constant of KA (ms)	KA-τ_A	0.454	0.42 to 0.7
6	Inactivation time constant of KA (ms)	KA-τ_I	6.54	3 to 10
7	$V_{1/2}$ activation of KA (mV)	KA-V_A	−55	−50 to −62
8	$V_{1/2}$ inactivation of KA (mV)	KA-V_I	−73.1	−69 to −82
**Delayed rectifier K$^+$ channel properties**				
9	Maximal conductance (μS/cm^2)	KDR-g	500	320 to 1100
10	Activation time constant of KDR (ms)	KDR-τ_A	6.4	5 to 10
11	$V_{1/2}$ activation of KDR (mV)	KDR-V_A	−44	−38 to −50
**Fast Na$^+$ channel properties**				
12	Maximal conductance (mS/cm^2)	Na-g	18	16 to 50
13	Activation time constant of Na (μs)	Na-τ_A	50	42 to 56
14	Inactivation time constant of Na (ms)	Na-τ_I	3	2 to 6
15	$V_{1/2}$ activation of Na (mV)	Na-V_A	−31	−30 to −40
16	$V_{1/2}$ inactivation of Na (mV)	Na-V_I	−49	−43 to −55
**Small conductance Ca^{2+} - dependent potassium (*SK*) channel properties**				
17	Maximal conductance (mS/cm^2)	SK-g	5	1 to 12
18	$Ca_{1/2}$ activation of SK (μM)	SK-C_A	4	1 to 8
19	Activation time constant of SK (ms)	SK-τ_A	214	195 to 250
20	Decay constant of calcium	Ca-τ_{decay}	160	95 to 206
**Large conductance Ca^{2+} - activated potassium (*BK*) channel properties**				
21	Maximal conductance (mS/cm^2)	BK-g	110	14 to 190
22	$C_{1/2}$ activation of BK (μM)	BK-C_A	4	2 to 7
23	Activation time constant of BK (Ca^{2+} dependent) (ms)	BK-$C\tau_A$	10	5 to 15
24	Activation time constant of BK (voltage dependent) (μs)	BK-τ_A	5	3 to 11
25	$V_{1/2}$ activation of BK (mV)	BK-V_A	−28	−18 to −36
***L*-type Ca^{2+} channel properties**				
26	Maximal conductance (μS/cm^2)	CaL-g	700	105 to 800
27	Activation time constant of L-type (μs)	CaL-τ_A	3	1 to 12
28	$V_{1/2}$ activation of L-type (mV)	CaL-V_A	−1.3	−5 to 7
***N*-type Ca^{2+} channel properties**				
29	Maximal conductance (μS/cm^2)	CaN-g	0.5	0.1 to 5
30	Activation time constant of N-type (ms)	CaN-τ_A	0.6	0.1 to 1
31	Inactivation time constant of N-type (ms)	CaN-τ_I	1297	1050 to 1450
32	$V_{1/2}$ activation of N-type (mV)	CaN-V_A	−21	−30 to −10
33	$V_{1/2}$ inactivation of N-type (mV)	CaN-V_I	−40	−50 to −30
***T*-type Ca^{2+} channel properties**				
34	Maximal conductance (μS/cm^2)	CaT-g	0.7	0.5 to 10
35	Activation time constant of T-type (ms)	CaT-τ_A	4	2 to 10
36	Inactivation time constant of T-type (ms)	CaT-τ_I	7665	6800 to 8400
37	$V_{1/2}$ activation of T-type (mV)	CaT-V_A	−36	−28 to −42
38	$V_{1/2}$ inactivation of T-type (mV)	CaT-V_I	−67	−75 to −58
**Passive properties**				
39	Specific membrane resistivity (kΩ.cm^2)	R_m	38	30 to 42
40	Specific membrane capacitance (μF/cm^2)	C_m	1	0.8 to 1.2

Supplementary Table S2. Parameters and their ranges for stochastic search of valid basket cells (Mishra and Narayanan, 2019)

	Parameter	Symbol	Default value	Testing range
***h* channel properties**				
1	Maximal conductance (μS/cm^2)	*h-g*	3	0.3 to 10
2	Activation time constant of I_h (ms)	h-τ_A	39	30 to 50
3	$V_{1/2}$ activation of I_h (mV)	h-V_A	–81	–70 to –90
***A*-type K+ Channel Properties**				
4	Maximal conductance (mS/cm^2)	*KA-g*	0.4	0.1 to 1.5
5	Activation time constant of KA (ms)	KA-τ_A	11.549	5 to 15
6	Inactivation time constant of KA (ms)	KA-τ_I	11.69	10 to 15
7	$V_{1/2}$ activation of KA (mV)	KA-V_A	–33	–28 to –38
8	$V_{1/2}$ inactivation of KA (mV)	KA-V_I	–83	–80 to –90
**Fast delayed rectifier K+ Channel Properties**				
9	Maximal conductance (S/cm^2)	*KDR-g*	0.0017	0.0011 to 0.0025
10	Activation time constant of KDR (ms)	KDR-τ_A	2.16	1 to 4
11	$V_{1/2}$ activation of KDR (mV)	KDR-V_A	–26.76	–20 to –30
**Na+ Channel Properties**				
12	Maximal conductance (mS/cm^2)	*Na-g*	200	90 to 300
13	Activation time constant of Na (ms)	Na-τ_A	0.066	0.055 to 0.075
14	Inactivation time constant of Na (ms)	Na-τ_I	3.99	2 to 8
15	$V_{1/2}$ activation of Na (mV)	Na-V_A	–29	–20 to –35
16	$V_{1/2}$ inactivation of Na (mV)	Na-V_I	–47.59	–40 to –55
**Passive Properties**				
17	Specific membrane resistivity (Ω.cm^2)	R_m	7100	5000 to 15000
18	Specific membrane Capacitance (μF/cm^2)	C_m	1	0.8 to 1.2
