# Supplementary material for: Ion-channel regulation of response decorrelation in a heterogeneous multi-scale model of the dentate gyrus
Source: Curr Res Neurobiol. 2021 Mar 5;2:100007. doi: 10.1016/j.crneur.2021.100007 (PMC7610774; doi:10.1016/j.crneur.2021.100007)
Supplement: Multimedia component 2 [file mmc2.pdf]

Peer Review Overview

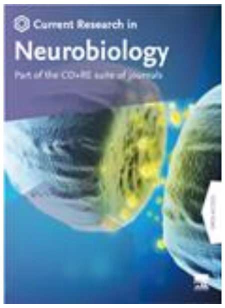

Manuscript Title: Ion-channel regulation of response decorrelation in a heterogeneous multi-scale model of the dentate gyrus

Received	Jul 27, 2020
1st Decision	Sep 29, 2020
1st Revision Submitted	Dec 28, 2020
Accepted	Feb 23, 2021

1st Decision letter

Reference: CRNEUR-D-20-00006
Title: Ion-channel regulation of response decorrelation in a heterogeneous multi-scale model of the dentate gyrus
Journal: Current Research in Neurobiology

Dear Dr. Narayanan,

Thank you for submitting your manuscript to Current Research in Neurobiology. Although the reviewers are generally supportive of the work the manuscript would require substantial revision to address all the points raised. In particular it would need to address the second reviewer's concern about the scientific advance and substantially improving the clarity of the paper. If you believe you are able to well address all of the raised concerns with a major revision please let us know and we will look forward to expecting the revised manuscript in the next 1-2 months. We are likely to need to consult the reviewers on whether the points have been addressed.

Thereby, I invite you to resubmit your manuscript after addressing the comments below. Please resubmit your revised manuscript by Nov 28, 2020.

When revising your manuscript, please consider all issues mentioned in the reviewers' comments carefully: please outline every change made in response to their comments and provide suitable rebuttals for any comments not addressed. Please note that your revised submission may need to be re-reviewed.

Current Research in Neurobiology values your contribution and I look forward to receiving your revised manuscript.

Kind regards,

Christopher I. Petkov
Editor in Chief
Current Research in Neurobiology

Comments from Editors and Reviewers:

Reviewer #1:

The authors of this manuscript address the question of how perturbations of individual ion channels contribute to changes in neuronal and network activity in the dentate gyrus, with a specific focus on a network-level computation called channel decorrelation. It is an ambitious project that seeks to identify how alterations in single ion channels alter neuronal activity across multiple scales of analysis from individual neurons to entire networks.

Using a conductance-based network model comprised of neurons that exhibit realistically-heterogeneous properties, they report that the overall impact of eliminating individual ion channels strongly depends on local heterogeneities (i.e. variability among neurons within the population). Thus, adult-born dentate neurons that are known to exhibit heterogeneous structure suppress the impact of ion channel perturbations on network decorrelation. In other words, the presence of neurogenesis confers functional robustness of network activity. This is a very novel idea about a physiological significance of adult neurogenesis in the dentate gyrus. This idea further has general relevance for understanding the potential significance of other types of "local heterogeneities" in neural networks that do not exhibit neurogenesis.

I appreciate the novelty of this analysis and the conclusions, and think these are appropriate questions to address using modeling due to the inherent complexities of the questions and analysis. I only have some minor clarifications and suggestions that may help make the work more accessible to a broader audience.

1) Introduction, paragraph 1. Since there are many pharmacological compounds that are extremely specific, I don't find this argument to be strong justification for the approach. I suggest deleting this part about pharmacological specificity, and perhaps focusing the justification on the complexities of the analysis itself on multiple scales.
"Experimental analyses of such cascades are rarely tested concurrently at multiple scales, and employing genetic manipulations of components is complicated by compensations consequent to the elimination of these components."

2). The authors provide a very clear explanation of the question that is addressed and its significance in the introduction, but that same clarity does not come through in the abstract that is too technical. I recommend that the authors re-write the abstract in a style that is more similar to the introduction. This is important to make the work appealing to a large audience of non-computational scientists interested in either cellular physiology or the dentate gyrus/neurogenesis. In particular, it is not clear that the work addresses a potential role of adult-born neurons in DG function until half-way through the abstract.

3) Condensing the list of "questions" in 3rd paragraph to focus on the primary questions of the role of local heterogeneities in cellular and network functions, and specifically how neurogenesis-induced structural heterogeneities alter DG network functions might highlight the main take-home messages of the work, as well as simplify the transition to the 4th paragraph.

4) Paragraph 5. A general audience is unlikely to understand the description of "many-to-many" and "all-to-all". Please define at first use (noting that these terms also complicate the abstract). At the end of the

paragraph, the meaning of the second half of the sentence beginning "Together, these results" is unclear because the role of neurogenesis-induced heterogeneities as a substrate for the expression of degeneracy has not been explained (this might fit better in the discussion).

6). Discussion. As noted, there are reported differences in ion channel expression (Mongiat et al., PLoS, 2009; Gonzalez et al., J Neurosci 2018) and Ca2+ handling (Stocca et al., J Physiol. 2007) that suggest young GCs are not simply a smaller version of mature GCs. There are also many simplifications in the model that are not mentioned, such as inclusion of only BC-type interneurons, lack of NMDARs that may provide non-linearities and oversimplifications of synaptic receptor kinetics. It understandable that such simplifications are required, but please also mention potential limitations of these.

7). Methods. Section iii. Is there a typo in the sentence starting "The diameters of GCs for the three distinct….." ? It is unclear what is meant by BC in the following text since the BC population is always mature "….fully immature (BC:1-3 μm)".

8). First sentence of the results seems to be a statement rather than a question.

9). In the graphical summary, perhaps the authors could include a cartoon of heterogeneously-sized GCs.

Reviewer #2:

The paper „Ion-channel regulation of channel decorrelation in a heterogeneous multi-scale model of the dentate gyrus" by Poonam Mishra and Rishikesh Narayanan is a natural continuation of their previous work. They have taken large complex computational models, explored the model space with stochastic parameter settings and perturbed these models by specific knock-outs. This type of approach has proven useful to explore networks and single cells. In the case here, the authors have explored the effects of perturbing single ion channels in single neurons on the specific functioning of the dentate gyrus microcircuit (multi-scale).

The conclusions of the work seem to be that perturbing ion channels in single neurons impacts the network and does it in a wide diversity of ways. To me, from a general perspective, this is not surprising since changing anything to a model will typically change its behaviour in different ways. The details are interesting here, but while reading the paper it does not become clear what one should focus on. I am missing the broader new insight that this approach yielded in this particular case. Maybe more exploration of the system would be useful to better extract a clear insight, or a stronger focus on one of the questions.

To me, this is very much reflected in the way the work is presented. The abstract is near unintelligible. It seems to be hard for the authors to extract the essence, maybe it requires some more time to think about this. I believe the manuscript needs to be rewritten. The abstract is too long and unintelligible, the Introduction section reiterates the concepts in the abstract without explanations and spends too much time on the findings in the present work compared to introducing the field and the particular questions of interest. The Methods section then introduces concepts and literature as well as describing the results but does not do a good job at presenting the methods in a manner that will allow others to reproduce them. In the Results section, in turn, too little time is spent on challenging the original model

and too much on the details of individual perturbations.

To sum it up: I am kind of missing the main message here, what are the principles on which these effects are based?

Other points:
- The overall model itself seems to be new and interesting but is not explored or introduced in any amount of detail here.
- You say heterogeneities are responsible for regulating the resilience of the DG, you should show this: Model with and without heterogeneities.
- You say adult born neurons may improve the robustness of the system, I think this can be the main story of your paper but the details of this should be worked out and you really need to show why and how strong the effect is (Figure 10A is not enough)
- Ambiguity between information channel and ion channel, also in abstract and title.
- Is a model with GCs and BCs enough? How do the results scale with neuron numbers?
- Figure 4 and others -> supplement, otherwise you lose yourself in details.
- Isn't it unrealistic to assume that knocking out individual ion channels will not lead to the recruitment of compensatory mechanisms at the level of the ion channel composition in single neurons but also at the level of the network?

1st Author Response Letter

Response to comments from Editors and Reviewers:

We thank the reviewers for their time and effort in assessing our manuscript and their positive, constructive and helpful comments. We gratefully thank them for their critical review and insightful comments on our study and the manuscript. In response to these constructive comments, we have performed several sets of additional experiments (included in the manuscript as new panels in Figure 8) and have made significant changes to the entire manuscript specifically addressing the reviewers' concerns. We believe that these changes overall have improved and strengthened the paper, apart from enhancing the readability. Grateful thanks to the reviewers!

As the new simulations, analyzing neurogenesis-driven structural heterogeneities, were performed with a larger network and involved several ion channel knockouts, their completion along with the associated analyses took longer than we had anticipated. A significant amount of time was spent towards tuning this network with scaled inputs, to account for the several lines of experimental evidence that the synaptic connectivity to immature neurons are low, and that this low connectivity counterbalances their high excitability (Mongiat et al., 2009; Dieni et al., 2013; Dieni et al., 2016; Li et al., 2017). We gratefully thank the editor for the extension that they had provided in completing these simulations and the associated analyses.

In what follows, we have provided point-by-point responses to comments from the reviewers. Please note that text in blue refers to reviewers' comments, and text in normal typeface corresponds to our replies.

Comments from Reviewer 1

The authors of this manuscript address the question of how perturbations of individual ion channels contribute to changes in neuronal and network activity in the dentate gyrus, with a specific focus on a network-level computation called channel decorrelation. It is an ambitious project that seeks to identify how alterations in single ion channels alter neuronal activity across multiple scales of analysis from individual neurons to entire networks.

Using a conductance-based network model comprised of neurons that exhibit realistically-heterogeneous properties, they report that the overall impact of eliminating individual ion channels strongly depends on local heterogeneities (i.e. variability among neurons within the population). Thus, adult-born dentate neurons that are known to exhibit heterogeneous structure suppress the impact of ion channel perturbations on network decorrelation. In other words, the presence of neurogenesis confers functional robustness of network activity. This is a very novel idea about a physiological significance of adult neurogenesis in the dentate gyrus. This idea further has general relevance for understanding the potential significance of other types of "local heterogeneities" in neural networks that do not exhibit neurogenesis.

I appreciate the novelty of this analysis and the conclusions, and think these are appropriate questions to address using modeling due to the inherent complexities of the questions and analysis. I only have some minor clarifications and suggestions that may help make the work more accessible to a broader audience.

We sincerely thank the reviewer for their time and effort in reviewing our manuscript. We thank the reviewer for their positive and constructive comments, and for highlighting the novelty of the underlying study and the conclusions. We also thank them for the specific pointers to make the the study more accessible to a broader audience. In what follows, we have provided point-by-point responses to the reviewer's comments and have rewritten parts of the manuscript in addressing them.

1) Introduction, paragraph 1. Since there are many pharmacological compounds that are extremely specific, I don't find this argument to be strong justification for the approach. I suggest deleting this part about pharmacological specificity, and perhaps focusing the justification on the complexities of the analysis itself on multiple scales. "Experimental analyses of such cascades are rarely tested concurrently at multiple scales, and employing genetic manipulations of components is complicated by compensations consequent to the elimination of these components."

We thank the reviewer for their comment, and we agree that the complexity motivations are stronger. We have removed the pharmacological specificity argument. The specific sentence mentioned by the reviewer has now been replaced by:

"The complexity involved in the assessment of such multi-scale cascades is enormous, owing to the disparate forms of biological heterogeneities inherent to the different network components and the intricate interactions between these distinct components that govern network function."

2) The authors provide a very clear explanation of the question that is addressed and its significance in the introduction, but that same clarity does not come through in the abstract that is too technical. I recommend that the authors re-write the abstract in a style that is more similar to the introduction. This is important to make the work appealing to a large audience of non-computational scientists interested in either cellular physiology or the dentate gyrus/neurogenesis. In particular, it is not clear that the work addresses a potential role of adult-born neurons in DG function until half-way through the abstract.

We thank the reviewer for their suggestion. We have now rewritten the abstract, specifically focusing on appeal to a larger audience. We have also emphasized our focus on adult neurogenesis and local heterogeneities throughout the abstract of the revised manuscript.

3)Condensing the list of "questions" in 3rd paragraph to focus on the primary questions of the role of local heterogeneities in cellular and network functions, and specifically how neurogenesis-induced structural heterogeneities alter DG network functions might highlight the main take-home messages of the work, as well as simplify the transition to the 4th paragraph.

We thank the reviewer for pointing this to us. We have considerably condensed the third paragraph focusing specifically on the principal questions, rather than all the questions. We have also emphasized neurogenesis-induced heterogeneities, both from the structural standpoint and the ability of adult neurogenesis to drive afferent network connectivity from the entorhinal cortex.

4) Paragraph 5. A general audience is unlikely to understand the description of "many-tomany" and "all-to-all". Please define at first use (noting that these terms also complicate the abstract). At the end of the paragraph, the meaning of the second half of the sentence beginning "Together, these results" is unclear because the role of neurogenesis-induced heterogeneities as a substrate for the expression of degeneracy has not been explained (this might fit better in the discussion).

We have considerably reduced this paragraph of the introduction. In the abstract and in the introduction, we have now expanded on what we intend to convey by many-to-many and allto-all mappings. We thank the reviewer for pointing this to us.

6). Discussion. As noted, there are reported differences in ion channel expression (Mongiat et al., PLoS, 2009; (Gonzalez et al., J. Neuroscience, 2018) and Ca2+ handling (Stocca et al., J Physiol. 2007) that suggest young GCs are not simply a smaller version of mature GCs. There are also many simplifications in the model that are not mentioned, such as inclusion of only BC-type interneurons, lack of NMDARs that may provide non-linearities and oversimplifications of synaptic receptor kinetics. It understandable that such simplifications are required, but please also mention potential limitations of these.

We thank the reviewer for their comment, and for recognizing the need for such simplifications. We incorporated adult neurogenesis into the DG network accounting for three changes: (i) structural changes in neurons reflecting reduced surface area of granule cells thereby matching the increased excitability of immature cells (van Praag et al., 2002; Aimone et al., 2014); (ii) reduction of the overall afferent drive to neurons based on their surface area, so that reduced drive in immature neurons

counterbalanced their high excitability (Mongiat et al., 2009; Dieni et al., 2013; Dieni et al., 2016; Li et al., 2017); and (iii) the orthogonal afferent connectivity, actively driven by adult neurogenesis (Aimone et al., 2006, 2009; Li et al., 2017; Lodge and Bischofberger, 2019; Luna et al., 2019), was incorporated as afferent heterogeneities into the network model. We had assumed that the other components, including those referred by the reviewer, do not change towards reducing computational complexity. We also did not incorporate other DG interneurons or NMDARs into our analyses. As suggested by the reviewer, we have now mentioned these as limitations in the last subsection of the Discussion section.

7). Methods. Section iii. Is there a typo in the sentence starting "The diameters of GCs for the three distinct….." ? It is unclear what is meant by BC in the following text since the BC population is always mature "….fully immature (BC:1-3 µm)".

We thank the reviewer for pointing us to this slip. The last part should have just been "(2–9 µm)". This has now been rectified.

8). First sentence of the results seems to be a statement rather than a question.

We thank the reviewer for pointing us to this slip. We have now rectified this.

9). In the graphical summary, perhaps the authors could include a cartoon of heterogeneously-sized GCs.

We have updated the graphical summary, which now shows GCs with different sizes. We have also introduced specific labels in the graphical summary pointing to the color code employed for granule cells vs. basket cells. We thank the reviewer for pointing this to us.

Comments from Reviewer 2

The paper „Ion-channel regulation of channel decorrelation in a heterogeneous multi-scale model of the dentate gyrus" by Poonam Mishra and Rishikesh Narayanan is a natural continuation of their previous work. They have taken large complex computational models, explored the model space with stochastic parameter settings and perturbed these models by specific knock-outs. This type of approach has proven useful to explore networks and single cells. In the case here, the authors have explored the effects of perturbing single ion channels in single neurons on the specific functioning of the dentate gyrus microcircuit (multi-scale).

We sincerely thank the reviewer for their time and effort in reviewing our manuscript. We thank the reviewer for their positive and constructive comments, and for highlighting the utility of the underlying methodology. We also thank them for the specific pointers to make the manuscript more readable. In what follows, we have provided point-by-point by responses to the reviewer's comments and have rewritten parts of the in addressing them.

The conclusions of the work seem to be that perturbing ion channels in single neurons impacts the network and does it in a wide diversity of ways. To me, from a general perspective, this is not surprising since changing anything to a model will typically change its behaviour in different ways. The details are interesting here, but while reading the paper it does not become clear what one should focus on. I am missing the broader new insight that this approach yielded in this particular case. Maybe more exploration of the system would be useful to better extract a clear insight, or a stronger focus on one of the questions.

We thank the reviewer for their comment. Broadly, we would like to emphasize the following as the novel contributions and the new insights of our study:

1. Methodological novelty: Please note that this study constitutes the first that systematically assesses the cascading impact of eliminating individual ion channels (at the molecular scale) on response decorrelation (a network scale functional outcome) in the dentate gyrus (DG). We performed this by systematically incorporating four distinct biological heterogeneities into the DG network. In doing this, we systematically spanned the scales of analyses from the molecular scale (ion channels), through the cellular scale (neuronal physiology) to the network scale (decorrelation). As response decorrelation has always been studied from a network-perspective, the impact of individual ion channels and local heterogeneities on response decorrelation has remained unexplored. Our study fills this lacuna through the use of a conductance-based multi-scale model for a DG network, and more importantly endowed with different forms of biological heterogeneities. The choice of methodology employed here provides us insights about the roles of individual ion channels in regulating network decorrelation, in the face of physiological and pathological perturbations.

2. Conceptual novelty of the findings: First, we demonstrate that the mapping between structural components and functional outcomes is many-to-many, across scales of DG function. Specifically, we show that many ion channels can alter any of the several single-neuronal electrophysiological measurement or network function (excitability and channel decorrelation); and, perturbation in any given ion channel altered several of these functions. Second, we show that individual ion channels play a pivotal role in altering channel decorrelation, in a manner that was dependent on the specific local and afferent heterogeneities. Third, our results unveil the importance of local heterogeneities, 6 especially of neurogenesis-induced structural properties, in maintaining functional resilience in the face of large pathological insults involving channelopathies. Finally, we show that the impact of eliminating individual ion channels on channel decorrelation is invariant to the specific values of input correlation. We would like to emphasize that the importance of local heterogeneities in maintaining functional resilience in the face of large changes to ion-channel perturbations, and the invariance of the impact of eliminating individual ion channels to the specific values of input correlation are especially novel contributions of this study.

3. Implications of our novel findings: Physiologically, the dentate gyrus has been demonstrated to be the lynchpin for two important functions of the hippocampus. The first implicates neuronal scale plasticity in DG neurons to engram formation, and the second assigns specific roles for the DG to response decorrelation. Our study is the first that demonstrates the impact of alterations in ion channels (as the mediator of neuronalscale plasticity during engram formation) to response decorrelation, thereby linking these two functional roles. From a pathophysiological standpoint, several neurological disorders have been linked to channelopathies in the DG, implying large

perturbations to channel densities and intrinsic excitability. Our study quantitatively demonstrates that these channelopathies could have significant impact on the ability of the DG network to perform response decorrelation. Importantly, our study also unveils the ability of local heterogeneities to confer resilience of network function to such channelopathy. These results imply that adult neurogenesis could play a pivotal role in maintaining functional robustness under physiological or pathological perturbations.

Broadly, the conclusions of single ion channel knockout (KO) simulations at single neuron level are broadly consistent with existing literature from other neurons (despite being specific to the ion channels expressed in DG neurons, and their signature electrophysiological properties. However, the impact of single ion channel KOs on network-scale function has not been assessed in the DG literature, and the analyses performed here provide novel insights on DG function (as mentioned above). The fundamental question here is on perturbations, and the role of local heterogeneities in regulating the impact to such perturbations. We submit that this is the first computational model on response decorrelation that accounts for biological heterogeneities in addressing this important question on how molecular-scale perturbations alter network function. This necessitated the complexity of conductance-based models, and the need to systematically account for the four distinct heterogeneities. These analyses showed that biological heterogeneities, beyond forming a substrate for degeneracy in the emergence of channel decorrelation (Mishra and Narayanan, 2019), also provide a substrate for functional resilience in the face of perturbations.

We thank the reviewer for raising this. This has definitely helped us to reframe the presentation of the manuscript, and emphasize the novel conclusions in this study in the context of existing literature. The revised manuscript explicitly incorporates these points to delineate the novel conclusions of the study.

To me, this is very much reflected in the way the work is presented. The abstract is near unintelligible. It seems to be hard for the authors to extract the essence, maybe it requires some more time to think about this. I believe the manuscript needs to be rewritten. The abstract is too long and unintelligible, the Introduction section reiterates the concepts in the abstract without explanations and spends too much time on the findings in the present work compared to introducing the field and the particular questions of interest. The Methods 7 section then introduces concepts and literature as well as describing the results but does not do a good job at presenting the methods in a manner that will allow others to reproduce them. In the Results section, in turn, too little time is spent on challenging the original model and too much on the details of individual perturbations. To sum it up: I am kind of missing the main message here, what are the principles on which these effects are based?

We thank the reviewer for systematically pointing to the specific lacunae in the presentation of our manuscript. We have made an effort to change every point mentioned by the reviewer in each section:

Abstract: We have now removed the many jargons that were present in the previous version. We have rewritten the abstract to emphasize the central question addressed here, highlighted (as suggested by the reviewer) the sequential traversal from ion channels through neurons to networks. The abstract also has been refocused towards conveying the main messages of the study, rather than

providing all the details.

Introduction: The jargons in the introduction have been removed. More references have been added to emphasize the link to pertinent literature and the questions addressed here. The paragraph that outlined the questions addressed here has been considerably, focusing specifically on the principal question addressed here (similar to the abstract). Finally, the last paragraph in the introduction on the present work has now been considerably reduced in size.

Methods: We have now expanded the methods section incorporating all the details that are essential for reproducing all aspects of the work.

Results: Although we have retained the details of individual perturbations, we have reduced redundancy wherever possible. Please note that we have placed the conclusions in the context of the literature in the discussion section, but not in results. We have also split large subsections in the previous version of the manuscript to smaller ones in the revised mansucript, towards improving readability.

Other points

The overall model itself seems to be new and interesting but is not explored or introduced in any amount of detail here.

We thank the reviewer for their positive comment on the novelty of the methodology. We have now updated the methods section to incorporate all the required details. More specifically, towards emphasizing the rigor associated with the overall methodology and the experimental design of the simulations, we have explained the rationale behind each step in the revised Methods section.

You say heterogeneities are responsible for regulating the resilience of the DG, you should show this: Model with and without heterogeneities.

Please note that our conclusions were derived specifically from neurogenesis-induced structural heterogeneities being responsible for regulating the resilience. We have performed simulations with ("Network with GCs of heterogeneous age") and without ("Network with mature GC population") structural heterogeneities (Figures 4–8 in the revised manuscript).

In addition, in the revised manuscript, we have added a new set of simulations involving a larger 575-neuron network that incorporated age heterogeneity as well. We had earlier presented simulation outcomes only for mature vs. immature neuronal population for the 575- neuron network. Based on the reviewer's suggestion, we have now incorporated an addition set that involved age heterogeneitty. We thank the reviewer for raising this point.

You say adult born neurons may improve the robustness of the system, I think this can be the main story of your paper but the details of this should be worked out and you really need to show why and

how strong the effect is (Figure 10A is not enough).

To strengthen our specific conclusions on the impact of structural heterogeneities in functional robustness that we had reported in Fig. 10 (which is Fig. 8 in the revised manuscript), we performed an additional set of computationally expensive simulations with the larger network (575 neurons), incorporating age-heterogeneity as well. The results reported in Fig. 8 account for populations from networks that are endowed with fully mature, fully mature and heterogenous age neurons. These additional simulations took time because the afferent inputs had to be scaled to account for the several lines of experimental evidence that the synaptic connectivity to immature neurons are low, and that this low connectivity counterbalances their high excitability (Mongiat et al., 2009; Dieni et al., 2013; Dieni et al., 2016; Li et al., 2017). In addition, the simulation of conductance-based network of 575 neurons spanning 1000 s of total simulation time for the virtual animal traversal (with a 25 µs integration time) with different ion channel eliminations made this computationally expensive.

Employing these simulations, we found that the robustness observed in Fig. 7 with the small network also extended to heterogeneous networks with larger neuronal population (Fig. 8). Specifically, we noted that the disruption to channel decorrelation with removal of individual ion-channels was larger in the network with mature GCs but was suppressed in the additional presence of immature neurons as well (Fig. 8).

We thank the reviewer for raising this point.

Ambiguity between information channel and ion channel, also in abstract and title.

We thank the reviewer for point this potential ambiguity. Yes, it was indeed difficult to present this study because the same word is used to represent transmembrane proteins (ion channels) and information pathways (channel decorrelation). We have tried our best to delineate these by distinguishing these terms (and the context of their usage) as much as possible, but owing to historic baggage of these phrases, it was impossible to avoid some amount of confusion.

To remove some of this ambiguity, we have now replaced “channel decorrelation” by the more general “response decorrelation” in the title and the abstract. However, we have retained the phrase “channel decorrelation” in the rest of the manuscript to distinguish between pattern and channel decorrelation.

We have consistently employed “ion channel” to represent the transmembrane proteins, and “channel decorrelation” for the physiological measurement. We noticed some instances 9 where ion channels were referred to simply as channels, and have rectified this in the revised manuscript.

Is a model with GCs and BCs enough?

Although GCs and BCs are the principal classes of cells in the DG, there are indeed other cell types and have been implicated in response decorrelation. We agree that the model could be expanded to incorporate other cell-types and heterogeneities associated with them as well, and have mentioned

this as a limitation of our study and as a potential future direction in the discussion section. We thank the reviewer for pointing us to this.

How do the results scale with neuron numbers?

With the addition of new panels in Fig. 8, we have repeated our analyses with two networks with different numbers of neurons (Fig. 7: 100 GC and 15 BC; Fig. 8: 500 GC and 75 BC). Owing to the computational complexity of the conductance-based network, endowed with four distinct forms of heterogenities requiring 1000 s simulation with a 25 μs integration period for each ion channel, simulating larger networks was impossible with the computational power that we had access to. Thus, we have shown that the results hold with two different network sizes. We thank the reviewer for raising this question.

Figure 4 and others -> supplement, otherwise you lose yourself in details.

We agree with the reviewer, and thank them for pointing this to us. We have now moved Figure 4 from the previous version of the manuscript to the supplementary. In addition, we have also moved Figures 7 and 11 from the previous version to the supplementary. As a consequence, there are now 8 main figures and 3 supplementary figures in the revised version of the manuscript.

Isn't it unrealistic to assume that knocking out individual ion channels will not lead to the recruitment of compensatory mechanisms at the level of the ion channel composition in single neurons but also at the level of the network?

We agree that there could be active compensations in the biological network in response to elimination of individual channels. However, our model does not account for active compensations towards achieving homeostasis, and the analysis here focuses on the acute impact of ion channel perturbation. We have now mentioned this in the discussion section pointing to frameworks (e.g., (O'Leary et al., 2013; O'Leary et al., 2014; Srikanth and Narayanan, 2015; O'Leary, 2018)) through which such incorporation could be made in future models. We thank the reviewer for raising this point.

REFERENCES

Aimone JB, Wiles J, Gage FH (2006) Potential role for adult neurogenesis in the encoding of time in new memories. Nat Neurosci 9:723-727.

Aimone JB, Wiles J, Gage FH (2009) Computational influence of adult neurogenesis on memory encoding. Neuron 61:187-202.

Aimone JB, Li Y, Lee SW, Clemenson GD, Deng W, Gage FH (2014) Regulation and function of adult neurogenesis: from genes to cognition. Physiol Rev 94:991- 1026.

Dieni CV, Nietz AK, Panichi R, Wadiche JI, Overstreet-Wadiche L (2013) Distinct determinants of sparse activation during granule cell maturation. J Neurosci 33:19131-19142. Dieni CV, Panichi R, Aimone JB, Kuo CT, Wadiche JI, Overstreet-Wadiche L (2016) Low excitatory innervation balances high intrinsic excitability of immature dentate neurons. Nature communications 7:11313.

Li L, Sultan S, Heigele S, Schmidt-Salzmann C, Toni N, Bischofberger J (2017) Silent synapses generate sparse and orthogonal action potential firing in adult-born hippocampal granule cells. eLife 6.

Lodge M, Bischofberger J (2019) Synaptic properties of newly generated granule cells support sparse coding in the adult hippocampus. Behav Brain Res 372:112036.

Luna VM, Anacker C, Burghardt NS, Khandaker H, Andreu V, Millette A, Leary P, Ravenelle R, Jimenez JC, Mastrodonato A, Denny CA, Fenton AA, Scharfman HE, Hen R (2019) Adult-born hippocampal neurons bidirectionally modulate entorhinal inputs into the dentate gyrus. Science 364:578-583.

Mishra P, Narayanan R (2019) Disparate forms of heterogeneities and interactions among them drive channel decorrelation in the dentate gyrus: Degeneracy and dominance. Hippocampus 29:378-403.

Mongiat LA, Esposito MS, Lombardi G, Schinder AF (2009) Reliable activation of immature neurons in the adult hippocampus. PloS one 4:e5320.

O'Leary T (2018) Homeostasis, failure of homeostasis and degenerate ion channel regulation. Curr Opin Physiol 2:129-138.

O'Leary T, Williams AH, Caplan JS, Marder E (2013) Correlations in ion channel expression emerge from homeostatic tuning rules. Proc Natl Acad Sci U S A 110:E2645-2654.

O'Leary T, Williams AH, Franci A, Marder E (2014) Cell types, network homeostasis, and pathological compensation from a biologically plausible ion channel expression model. Neuron 82:809-821.

Srikanth S, Narayanan R (2015) Variability in State-Dependent Plasticity of Intrinsic Properties during Cell-Autonomous Self-Regulation of Calcium Homeostasis in Hippocampal Model Neurons. eNeuro 2:ENEURO.0053-0015.2015.

van Praag H, Schinder AF, Christie BR, Toni N, Palmer TD, Gage FH (2002) Functional neurogenesis in the adult hippocampus. Nature 415:1030-1034.

Accept Letter

Dear Dr. Narayanan,

Thank you for submitting your manuscript to Current Research in Neurobiology.

I am pleased to inform you that your manuscript has been accepted for publication.

Please ensure that the remaining corrections are made at the proofing stage.

If you are interested in an Author Q&A, we would be happy to send you some questions to answer to help to better engage with the public around your research. Please let us know if you are interested and if possible pleaes provide a picture of the first and last author.

Your accepted manuscript will now be transferred to our production department. We will create a proof which you will be asked to check, and you will also be asked to complete a number of online forms required for publication. If we need additional information from you during the production process, we will contact you directly.

We appreciate you submitting your manuscript to Current Research in Neurobiology and hope you will consider us again for future submissions.

Kind regards,

Christopher I. Petkov
Editor in Chief
Current Research in Neurobiology

Editor and Reviewer comments:

Reviewer 1: The authors have addressed my prior concerns to improve the manuscript.

Reviewer 2: I agree with the authors in their response to my concerns when they emphasize the novelty of their approach. I am unfortunately still not entirely convinced that new robust insights come from this work. Differences at the network level are there as is to be expected if the individual components making up the networks are changed. But it seems to me that these differences are not entirely understood and that for example the differences between model conditions (mature, immature, heterogeneous) remain not obvious and depend strongly on the ion channels that are knocked out etc... However, it is fair to say that the authors did a significant revision and it is clear to the reader what exactly the authors have done. So, I do not object to publishing this work. I am sorry if I myself remain not entirely convinced. This may very well be a comprehension hurdle on my side.

I would suggest putting x and y axes in Figures 5AC and 6A in the same range to not confuse the reader or normalize the results adequately.

Also, I had asked for correcting the equations in the manuscript but in my current version most equations remain blanked out.

-------- End of Review Comments --------
